# Synergistic antipersister, efflux inhibitory & antibiofilm activities of vaginal Lactobacillus-derived postbiotics against UPEC: toward a novel therapeutic for utis

**DOI:** 10.1038/s41598-026-35736-7

**Published:** 2026-01-10

**Authors:** Veena G. Nair, David Raj Chellappan, Ramya Devi Durai, Rajesh Y.B.R.D, Dhiviya Narbhavi, Anupriya A., Prabhusaran N., Saisubramanian Nagarajan

**Affiliations:** 1 Antimicrobial Resistance Lab, Centre for Research in Infectious Diseases, School of Chemical and Biotechnology, SASTRA Deemed to be University, Thanjavur, 613401 Tamil Nadu India; 2 Central Animal House Facility, SASTRA Deemed to be University, Thanjavur, 613401 India; 3 Pharmaceutical Technology Laboratory, School of Chemical and Biotechnology, SASTRA Deemed to be University, Thanjavur, 613401 India; 4 Department of Chemistry, School of Chemical and Biotechnology, SASTRA Deemed University, Thanjavur, 613 401 Tamil Nadu India; 5 Department of Obstetrics and Gynaecology, TSRMMCH&RC, Tiruchirappalli, Tamil Nadu India; 6 Department of Microbiology, TSRMMCH&RC, Tiruchirappalli, Tamil Nadu India; 7 Research Faculty, Institutional Research Board TSRMMCH&RC, Tiruchirappalli, Tamil Nadu India

**Keywords:** Uropathogenic *E coli* persisters, Vaginal probiotics, Cell-free supernatant, Postbiotics, Anti-persister potential, Customised vaginal wash, Preclinical validation, Drug discovery, Microbiology

## Abstract

Bacterial persisters, phenotypic variants that can survive high doses of antibiotics, pose a significant challenge in treating chronic infections and contribute to post-treatment relapses. In this study, we investigated the impact of cell-free supernatant (CFS) from human vaginal *Lactobacillus* spp. on the formation of *E. coli* UTI89 persister cells. Among various antibiotics tested, colistin exposure resulted in the highest percentage of persister cells, followed by meropenem, with ampicillin showing the least effect. Fractionation of the CFS via column chromatography yielded seven fractions, with fractions C4 and C7 demonstrating a synergistic inhibition of *E. coli* persisters. Gas chromatography-mass spectrometry (GC-MS) identified itaconic anhydride and (-)-terpinen-4-ol as the key metabolites responsible for this effect. When combined with colistin and meropenem, these compounds (8 µg/ml and 5 µg/ml, respectively) significantly reduced persister cell formation. The bactericidal action was found to be ROS-dependent, as evidenced by the reduced efficacy in the presence of thiourea, a hydroxyl radical scavenger. Additionally, these metabolites increased membrane permeability and inhibited efflux pumps, further enhancing antibiotic efficacy. Building on these findings, we developed a novel postbiotic-based vaginal wash formulation incorporating (-)-terpinen-4-ol, itaconic anhydride, and tryptamine, another metabolite with antibiofilm properties. These bioactive compounds, isolated from indigenous human vaginal *Lactobacillus* spp., were incorporated into a poloxamer 407-based formulation designed to prevent urinary tract infections (UTIs) and combat antimicrobial resistance. Preclinical studies conducted on BALB/c mice models validated the formulation’s efficacy and safety, highlighting its potential for clinical translation. This approach of combining the antipersister, efflux inhibitory, and antibiofilm activities of these postbiotics within a practical therapeutic formulation offers a promising strategy for improving UTI management and combating the growing challenge of antibiotic tolerance.

**Supplementary Information:**

The online version contains supplementary material available at 10.1038/s41598-026-35736-7.

## Introduction

 UTIs are the most common bacterial infection that affects an estimated population of 150 million per year worldwide, including 7 million acute urinary tract infection cases^1^. Women exhibit a threefold higher incidence of UTIs (6.6%) compared to men (1.8%), with an average recurrence rate of 2.6 episodes per year^2^. Recurrent UTIs account for nearly 10.5 million outpatient visits and 2–3 million emergency visits annually, contributing to substantial morbidity, loss of productivity, and an estimated global economic burden of USD 3.5 billion per year^3^. Increasing antimicrobial resistance further exacerbates this problem. Reports from the European Centre for Disease Prevention and Control indicate high resistance rates in uropathogens, including 42% resistance to third-generation cephalosporins in *Escherichia coli* and 35% methicillin resistance in *Staphylococcus aureus* across multiple countries^4^. Notably, for urinary tract infections caused by *E. coli*, a troubling 1 in 5 cases exhibited reduced susceptibility to standard antibiotics like ampicillin, co-trimoxazole, and fluoroquinolones in 2020^5^, emphasising the urgent need for global strategies to address antibiotic resistance.

In recent years, growing interest has emerged in microbiome-based interventions aimed at restoring host–microbe balance rather than eradicating microorganisms indiscriminately^6^. Although probiotics are generally considered safe, their administration raises concerns in certain populations, including pregnant women, and presents challenges related to formulation stability, storage, and regulatory approval^7^. These limitations have driven increasing attention toward postbiotics, non-viable microbial cells and their bioactive metabolites, which retain health benefits while offering improved safety, stability, and reproducibility^8^. The International Scientific Association for Probiotics and Prebiotics (ISAPP) formally defined postbiotics in 2021 as preparations containing inanimate microorganisms and/or their components that confer health benefits to the host^9^. Lactobacillus species dominate the healthy vaginal microbiota and play a critical role in protecting against urogenital infections by maintaining an acidic vaginal pH (3.8–4.4) and producing antimicrobial compounds. These bioactive substances include lactic acid, hydrogen peroxide, bacteriocins, surfactants, and other metabolites, which effectively inhibit colonisation and overgrowth of vaginal and uropathogens^10^.

In our earlier work, we identified unique bioactive metabolites from human vaginal Lactobacillus isolates, including tryptamine and (-)-terpinen-4-ol, with potent antibiofilm and antimicrobial properties. Tryptamine was shown to disrupt the exopolysaccharide matrix of uropathogenic *E. coli* biofilms, compromising biofilm integrity^11^ (Fig. S1). Also, (-)-terpinen-4-ol demonstrated efflux pump inhibitory potential, restoring antibiotic susceptibility in multidrug-resistant *E. coli* and *Klebsiella pneumoniae*^12^ (Fig. S2).

A major challenge in the treatment of recurrent UTIs is the presence of bacterial persister cells—metabolically dormant subpopulations that exhibit multidrug tolerance without acquiring genetic resistance^13^. Persister cells survive antibiotic exposure, re-emerging upon removal of stress and driving chronic and recurrent infections. In uropathogenic *E. coli*, persistence is further reinforced by reduced metabolic activity and enhanced efflux mechanisms that limit intracellular antibiotic accumulation^14^.

Although several synthetic compounds and metabolic stimuli have been explored to eradicate persister cells, clinically viable and safe solutions remain limited^15^. In the present study, we identified a cell-free vaginal probiotic supernatant enriched with itaconic anhydride and (-)-terpinen-4-ol that synergistically inhibits the growth and survival of uropathogenic *E. coli* persister cells. Leveraging these findings, we developed a customised vaginal wash formulation incorporating these Lactobacillus-derived postbiotics, along with postbiotic tryptamine within a poloxamer 407 base, a biocompatible thermoresponsive polymer well suited for mucosal delivery. Preclinical evaluation in BALB/c mouse models demonstrated both the efficacy and safety of the formulation. This study presents a novel postbiotic-based vaginal therapeutic strategy aimed at preventing recurrent UTIs, addressing antimicrobial resistance, and improving women’s urogenital health.

## Results

### *E. coli UTI89* strain generates persister cells in response to different antibiotic classes

**Fig. 1:**
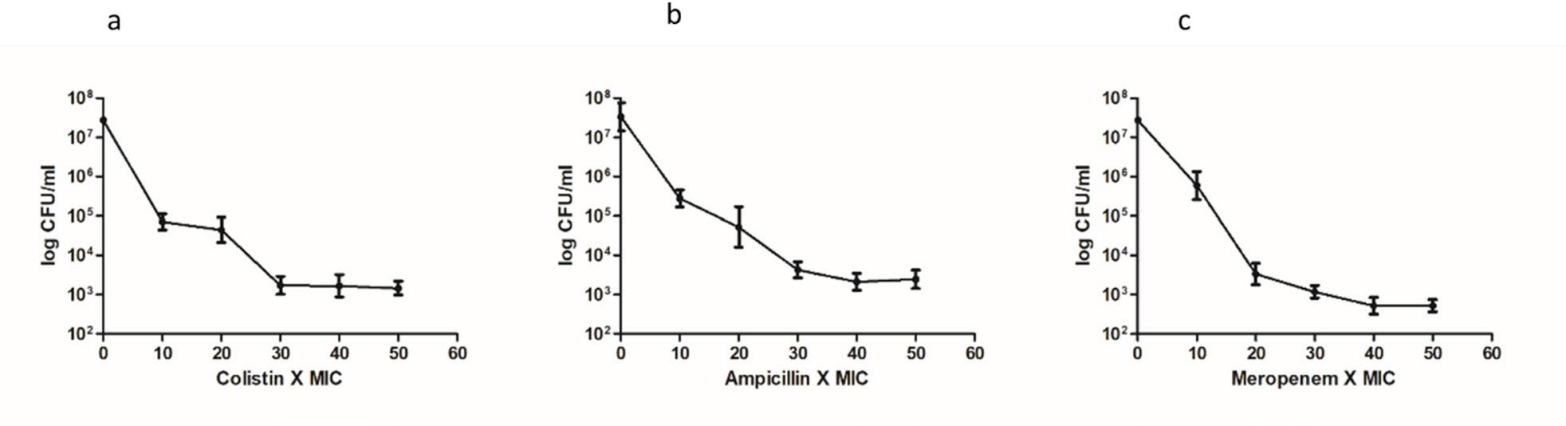
Dose-dependent Persister Assay. Persister cell formation on 30X to 40X MIC of antibiotics in the late exponential phase of *E. coli* UTI89 treated for 3 h with **a**) colistin **b**) Ampicillin **c**) Meropenem. Untreated cells used as the control had a cell count of 4.1 ± 1 × 10^9^ CFU/ml. Three independent experiments are represented by the data. Error bars represent the mean ± SD.

UPEC strain (*E. coli* UTI89) developed persister cells in response to 30X MIC of colistin (MIC 2 µg/ml; 30X MIC = 60 µg/ml), 40X MIC of Ampicillin (MIC 100 µg/ml;40X MIC = 400 µg/ml) and 40X MIC of Meropenem (MIC 4 µg/ml; 40X MIC = 160 µg/ml), when bacteria were in the late exponential phase of their growth (Fig. 1a, b and c). The time-dependent experiment showed that exposure of the population to 30X MIC of colistin resulted in 1.12% persisters, exposure to 40X MIC of meropenem resulted in 0.29% persisters, and 40X MIC of ampicillin generated 0.12% persisters. No regrowth was observed, and the persister cells, as expected, were not resistant to antibiotics (Fig. S3). The persister cells of *E. coli* UTI89 harbouring a Green Fluorescent Protein (GFP) expressing plasmid displayed a lack of growth in antibiotic-containing medium, while it showed growth in antibiotic-free medium, as evidenced by growth-induced turbidity and fluorescent imaging of a portion of the liquid medium, which confirms that the formed cells are persisters (Fig. S4 A-E). Growth of persister cells in the presence of CFS from *Lactobacilli* indicated that CFS did not exert an antimicrobial effect on persister cells (Fig. S4F). The typical biphasic decline in cell counts with a substantial decrease in drug-sensitive cells followed by a plateau of the surviving persister subpopulation revealed that the persister cells were developed at 12 h for *E. coli* UTI89 when treated with 30X MIC colistin, 40X MIC of Ampicillin and 40X MIC of meropenem, respectively (Fig. S5).

### Non-heritability of persister cells

To isolate persister cells and ascertain their nature, cultures in the late exponential phase were grown to a cell density of 10^7^ cfu/ml, treated with 30X and 40X MIC of antibiotics, and the cells were collected hourly for three hours. The cultures were rinsed and inoculated into fresh medium after 3 h of treatment. They were further grown up to the exponential phase to attain the same cell density of 10^7^ cfu/ml before the second and third antibiotic additions. These experiments were conducted three times consecutively, and there was no variation in the production of the persister cells, and the persister population remained almost constant (Fig. S6). The persister cells after each passage showed same tolerance to the antibiotic, in contrast to the parent culture, demonstrating the non-heritability of persistence with an increase in tolerance to antibiotics as reported earlier^16,17^.

### CFS/metabolites from Lactobacillus spp. Significantly reduces the development of *E. coli* UTI89 persister cells

Relative to treatment with antibiotics (30X/40X MIC) alone, CFS, along with 30X MIC of colistin, resulted in a significant 4.7 log reduction in persister cell formation. Similarly, CFS treatments along with 40X meropenem and 40X ampicillin exhibited substantial reductions in persister cell formation, with reductions of 4.2 log and 4 log, respectively (Fig. S7).

**Fig. 2:**
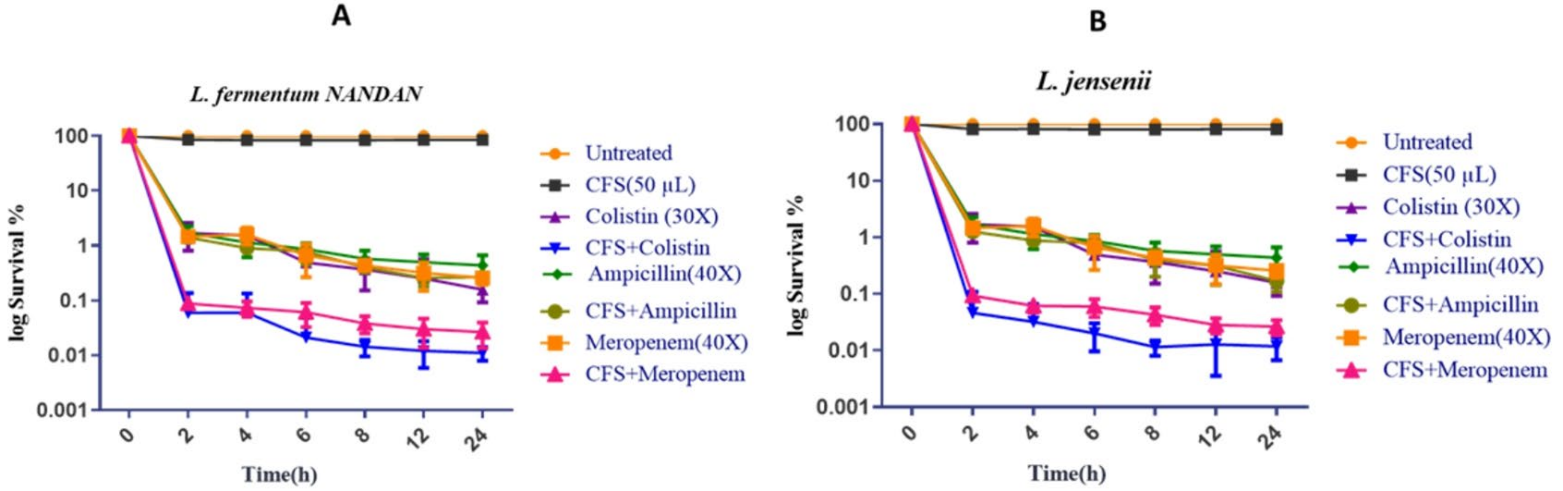
Effectiveness of different combinations of antibiotics and cell-free supernatants from *L. fermentum* and *L. jensenii* in reducing the persistence of *E. coli UTI89.* **A**) *L. fermentum NANDAN* **B**) *L. jensenii.* Untreated cells were used as a control. The data is representative of three independent experiments. Error bars represent the mean ± SD.

The time-dependent persister assay demonstrated that the combination of 50 µL of cell-free supernatant from *Lactobacillus fermentum* and *Lactobacillus jensenii*, along with antibiotics (Fig. 2A and B), exhibited the highest efficacy in reducing the persister cells of *E. coli* UTI89. The optimal reduction was observed with CFS in combination with colistin, followed by CFS along with meropenem. These observations imply the effectiveness of this specific combination in combating *E. coli UTI89* persistence.

### Screening and characterisation of the metabolites from CFS against persisters

**Fig. 3:**
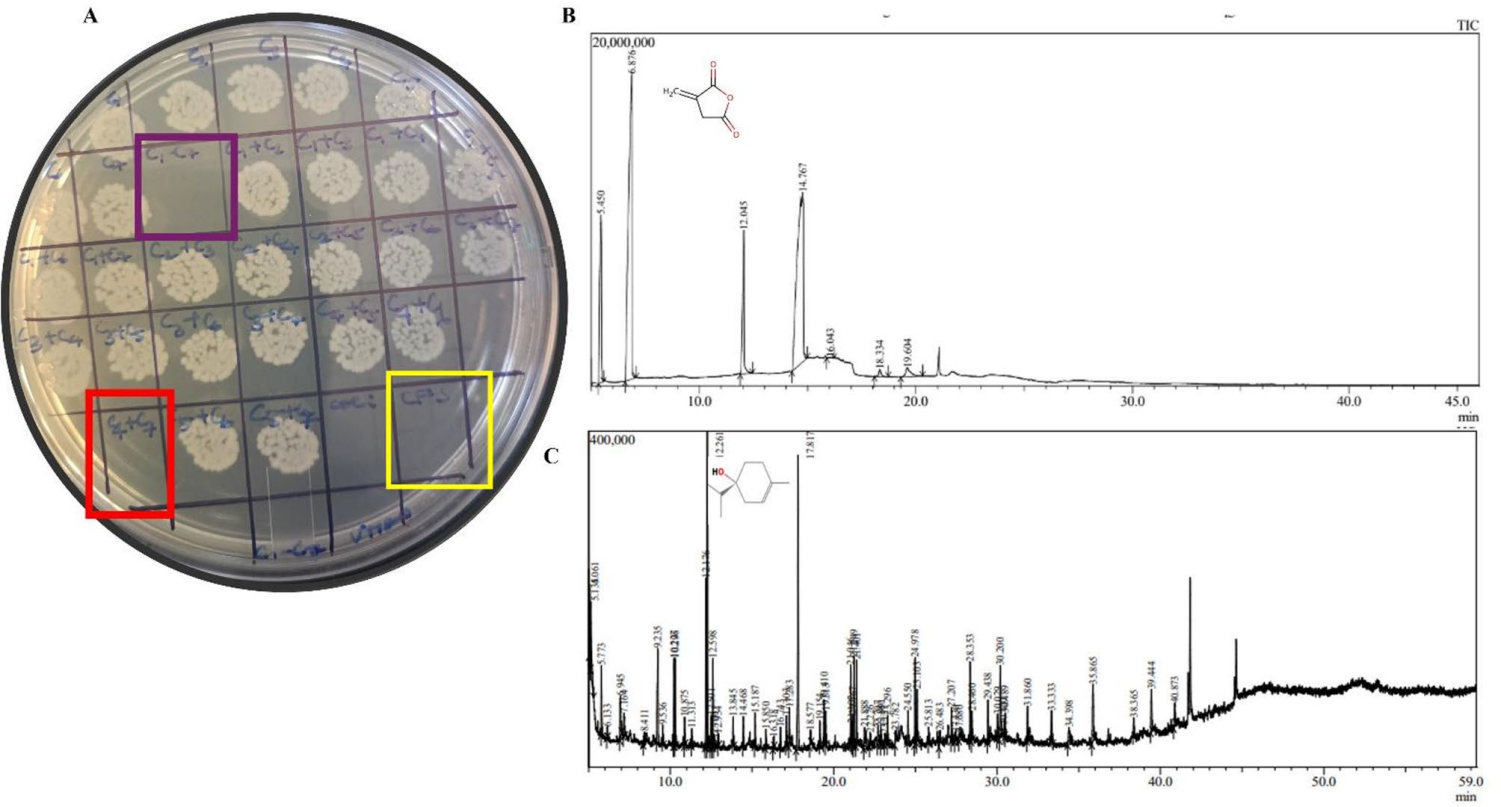
Screening and Characterisation of postbiotic metabolites. **A**) Colistin-derived persister cells were spotted, and purified fractions from CFS were applied alone and in combination. The combined effect of compounds C4 and C7 (highlighted in red) resulted in the absence of visible growth. Notably, the CFS combined with colistin (40x) also exhibited no growth, as indicated by the yellow-marked area. Similarly, when all compounds were added together, marked in purple, a striking absence of growth was observed. The data is representative of three independent experiments. Gas Chromatography-Mass Spectrometry (GC-MS) of **B**) Compound 4(C4), the highest peak with R. time 6.876 indicates the compound is Itaconic Anhydride. **C**) Compound 7(C7), the highest peak with R. time 12.261, indicates the compound is (-)-Terpinen-4-ol as confirmed by the NIST database.

CFS of *Lactobacillus* strains sourced from human vaginal microflora was subjected to bioassay-guided chromatographic separation to identify anti-persister compound(s) from among the postbiotics produced by *Lactobacilli.* Seven fractions (compounds) were isolated from the Cell-free supernatant. Notably, compounds C4 and C7 demonstrated synergistic inhibition of persister cells (Fig. 3A). To elucidate the chemical identities of these compounds, gas chromatography-mass spectrometry (GC-MS) analysis was employed, which revealed that compound C4 exhibited a significant peak with a maximal area of 181,182,264 units and a retention time of 6.876 min. This peak exhibited 97% similarity match to 2,5-Furandione, dihydro-3-methylene-, also known as Itaconic Anhydride, based on the National Institute of Standards and Technology (NIST) database (Fig. 3B). Similarly, compound C7 exhibited a dominant peak with a maximal area of 1,529,209 units with a retention time of 12.261 min. This peak demonstrated 94% similarity match to Cyclohexen-1-ol, 4-methyl-1-(1-methylethyl)-, (R)-, also referred to as (-)-Terpinen-4-ol, an isomer of terpineol (Fig. 3C).

### Synergistic combination of antibiotics and postbiotics enhances reactive oxygen species (ROS) production in *E. coli* UTI89 persister cells

**Fig. 4:**
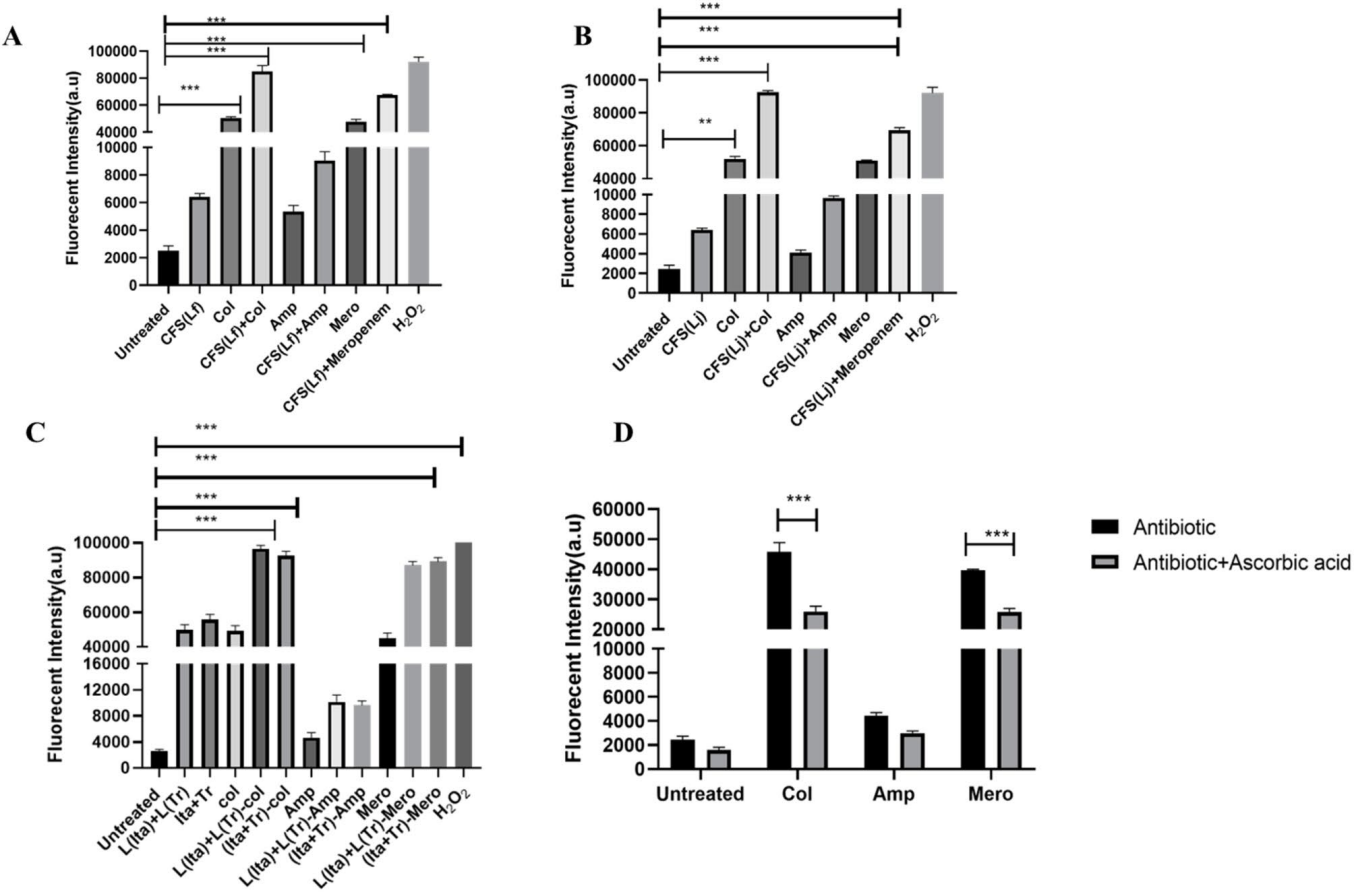
Effect of ROS on the persistence of the late exponential phase cells of *E. coli* UTI89. ROS levels in *E. coli* UTI89 upon treatment with **A**) 30X colistin (Col), 40X meropenem (Mero), 40X ampicillin (Amp) alone and in combination with Cell free supernatant (50 µL) of *L. fermentum* **(B)** 30X colistin (Col), 40X meropenem (Mero), 40X ampicillin (Amp) alone and in combination with Cell free supernatant (50 µL) of *L. jensenii.* **(C)** 30X colistin (Col), 40X meropenem (Mero), 40X ampicillin (Amp) alone and in combination with extracted (L(Ita) + L(Tr) and procured (Ita + Tr) Itaconic *anhydride and* (-)-Terpinen-4-ol **(D)** 30X colistin (Col), 40X meropenem (Mero), 40X ampicillin (Amp) alone and in combinations with antioxidant, ascorbic acid (100 µM). The data is representative of three independent experiments. Error bars represent the mean ± SD. *p ≤ 0.05; **p ≤ 0.01; ***p ≤ 0.001.

When colistin-derived Persister cells of *E. coli* UTI89 cells were treated with 30X MIC of colistin, a 15.2-fold increase in the induction of reactive oxygen species (ROS) relative to the untreated control was observed. Similarly, meropenem caused a 14.3-fold increase in ROS, and ampicillin induced a 1.52-fold increase in ROS. Intriguingly, postbiotic CFS from both *L. fermentum* and *L. jensenii* caused a 3-fold elevation in ROS levels relative to the untreated cells. When CFS was combined with antibiotics, ROS levels increased drastically to 25.16-fold with colistin, 23.44-fold with meropenem, and 4.33-fold with ampicillin (Fig. 4A and B). A combination of either extracted/commercially procured Itaconic Anhydride (8 µg/ml) and (-)-terpinen-4-ol (5 µg/ml), along with colistin, displayed ROS production that resembled CFS + colistin treatment. Interestingly, a combination of Itaconic Anhydride (8 µg/ml) and (-)-terpinen-4-ol (5 µg/ml), along with meropenem exhibited 1.5-fold elevated ROS levels relative to the combination of CFS + Meropenem (Fig. 4A and C) which could be attributed to the relative difference in concentration of metabolites in CFS and in pure form (Fig. 4C).

Treatment with ascorbic acid displayed antioxidant effects at a concentration of 100 µM by lowering ROS levels induced by colistin from 15.5 to 7.9-fold. Similarly, it reduced meropenem-induced ROS from 14.9 to 7.6-fold (Fig. 4D). A comparable effect was also seen upon the addition of thiourea at sub-inhibitory doses (200 mM), which reduced the production of ROS by 7.9-fold in both colistin and meropenem-treated groups. A combination of antibiotics (colistin/meropenem) and CFS from either *L. fermentum* or *L. jensenii*, along with sub-inhibitory thiourea, reduced ROS levels by 10.5-fold. A similar effect was observed upon treatment with a combination of either extracted/procured itaconic anhydride (8 µg/ml) and (-)-terpinen-4-ol (5 µg/ml) along with antibiotics (Fig. S8). These observations highlight the potential synergistic interaction between antibiotics and postbiotics in CFS in mitigating persisters through enhanced ROS production.

### Synergistic action of metabolites leads to membrane permeability *of E. coli* UTI89 persisters

**Fig. 5:**
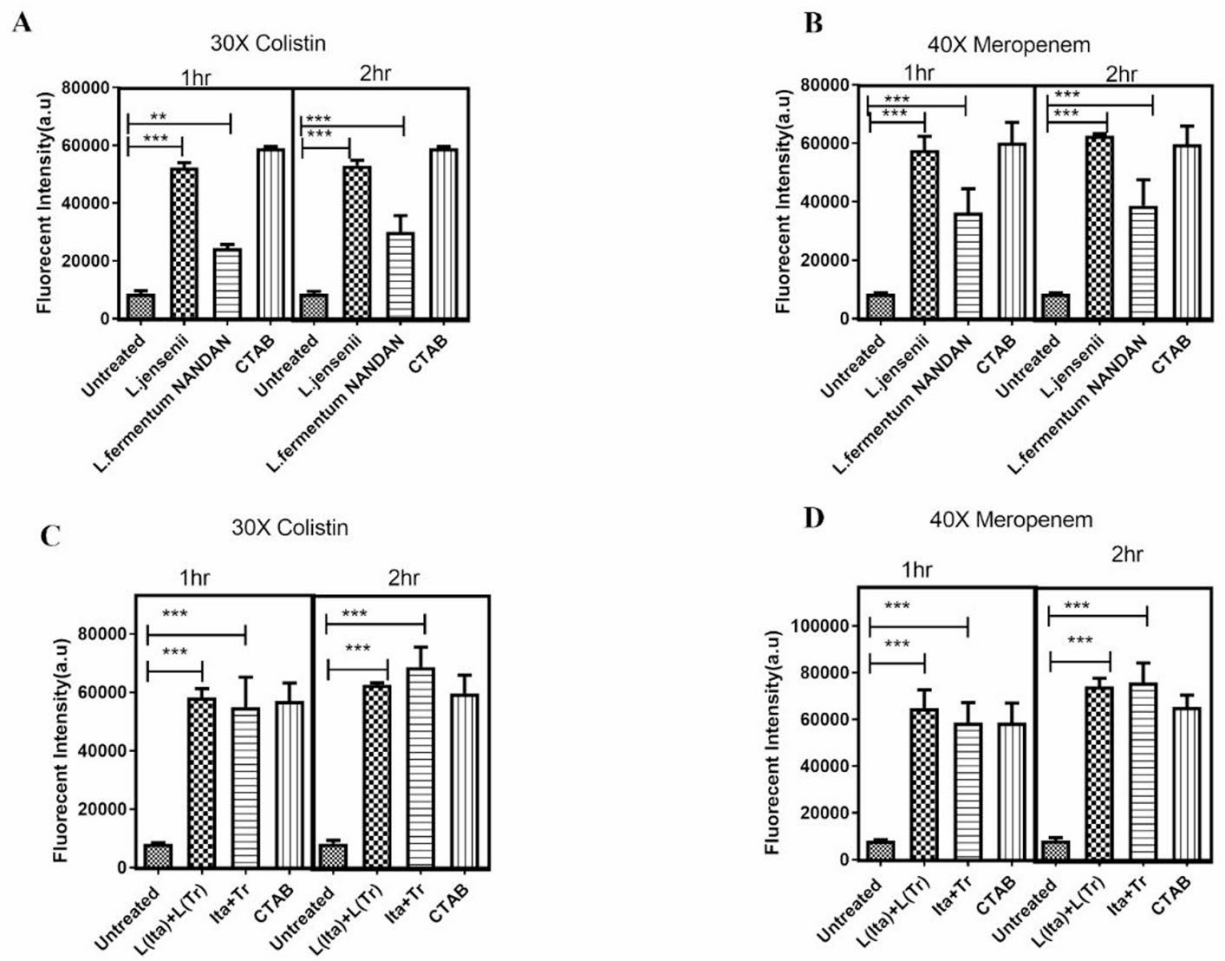
Effect of CFS of *L. jensenii* and *L. fermentum* on the outer membrane permeability of persister cells. NPN fluorescence intensity of A) colistin derived persister after exposure to CFS for 1 and 2 h.B) meropenem derived persister cells after exposure to CFS for 1 and 2 h C) colistin derived persister cells after exposure to combination of extracted itaconic anhydride (8 µg/ml) and (-)-terpinen-4-ol (5 µg/ml) (L(Ita) + L(Tr), procured compounds (Ita + Tr). D) meropenem derived persister cells after exposure to combination of either extracted itaconic anhydride (8 µg/ml) and (-)-terpinen-4-ol (5 µg/ml) (L(Ita) + L(Tr) or procured compounds (Ita + Tr). The data is representative of three independent experiments. Error bars represent the mean ± SD (**p < 0.001, ***p < 0.0001).

Colistin-derived persister cells were treated with CFS, and the membrane permeability of CFS-treated and untreated cells was compared. The fluorescence intensity of the probe NPN (N-phenyl-1-naphthylamine) was found to be significantly higher (4.3-fold increase, *p* < 0.0001) in *L. jensenii  *CFS treated cells compared to untreated cells. Similarly, *L. fermentum* NANDAN CFS led to t a 1.4-fold rise in membrane permeability (*p* < 0.001) indicating altered membrane permeability (Fig: 5 A). Exposure of meropenem derived persister cells to CFS from *L. jensenii* led to a 4.8-fold rise (*p* < 0.0001) in NPN fluorescence intensity relative to untreated cells (Fig: 5B). Similarly, CFS from *L. fermentum* NANDAN also caused 3.6-fold increase (*p* < 0.0001) in in NPN fluorescence intensity. Treatment with either extracted/purified itaconic anhydride (8 µg/ml) and (-)-terpinen-4-ol (5 µg/ml) also induced a fourfold increase in NPN fluorescence for both colistin and meropenem-treated cells implying (Fig. 5C and D) an elevated membrane permeability triggered by the postbiotic metabolites from the CFS of *Lactobacilli*, which can potentially lead to the leakage of intracellular components and compromise cellular integrity of persister cells.

## Scanning electron microscopy (SEM)

Colistin/Meropenem-derived persister cells of *E. coli* UTI89 cells were untreated or exposed to CFS/combination of either extracted or procured postbiotic metabolites, and were allowed to form biofilms on urinary catheters. The formed biofilms were fixed, dehydrated, and imaged using scanning electron microscopy (SEM). The SEM images of biofilms reveal distinct characteristics of persister cells formed after treatment with 30X colistin and 40X meropenem. The persister cells were observed to form thick biofilms composed of smooth cells with no surface perturbations (Fig. S9 A&C). However, upon treatment with the cell-free supernatant (CFS), the persister cells resulting from meropenem treatment (Fig. S9D) appear to exhibit round shape. The observed change in shape in meropenem-treated persister cells suggests a possible impact of the probiotic supernatant on cellular morphology. In contrast, the persister cells resulting from colistin treatment (Fig. S9B) display surface roughness and disruptions in membrane integrity, with visible pores compared to the untreated cells. After treatment with a combination of extracted itaconic anhydride (8 µg/ml) and (-)-terpinen-4-ol (5 µg/ml), similar effects were observed, further indicating the impact of the combined treatment on the morphology and integrity of persister cells (Fig. S9 E & F). Overall, the SEM images support the notion that the postbiotic metabolites from the cell-free vaginal probiotic supernatant has a significant modulatory effect on persister cell characteristics and biofilm formation, potentially contributing to the mitigation of UPEC persisters.

### Synergistic action of metabolites leads to efflux pump Inhibition of *E. coli* UTI89 persisters

Earlier studies have shown that efflux plays a role in the development or maintenance of persisters, especially in biofilms^18^. Hence, we explored whether reduced persister viability is associated with efflux inhibition mediated by CFS of *Lactobacilli* spp.

### EtBr accumulation

**Fig. 6:**
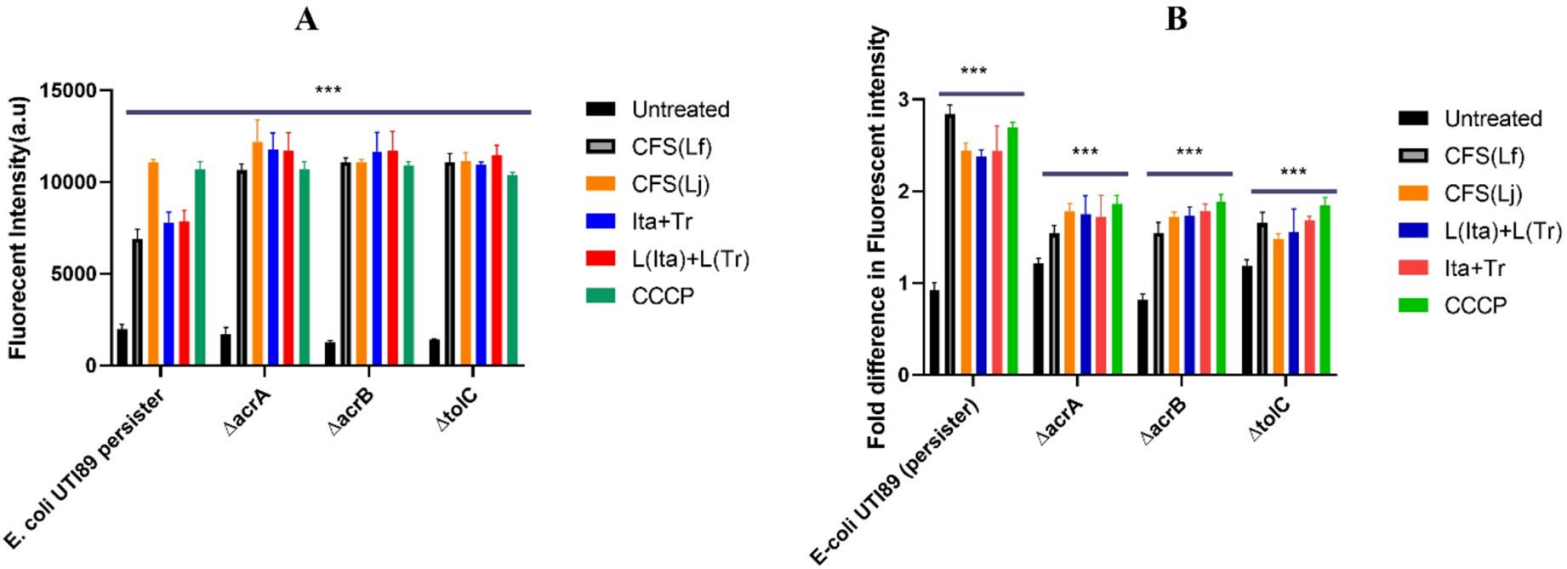
Postbiotic metabolites from *Lactobacilli* inhibits EtBr efflux in UTI89. The bar graph (**A**) presents the effects of different treatments on ethidium bromide (EtBr) accumulation in wild-type *E. coli UTI89* and pump-deficient *E. coli* mutants *(∆acrA*,* ∆acrB*,* and ∆tolC*). **B**) represents EtBr efflux in *E. coli UTI89/*pump mutants. Carbonyl cyanide 3-chlorophenylhydrazone (CCCP) was used as a positive control. Experiments were performed in triplicate and the data shown are the mean values with error bars indicating the standard deviation.

The study examined the effect of cell-free supernatants (CFS) from *Lactobacillus fermentum NANDAN* and *Lactobacillus jensenii* as well as a purified metabolite combination of terpinen-4-ol and itaconic anhydride, on the accumulation of ethidium bromide (EtBr) in *E. coli* UTI89 persister cells. It was observed that the addition of CFS from both strains significantly increased the accumulation of ethidium bromide (EtBr) in the cells. The increase in EtBr accumulation was 5.2-fold for *L. fermentum NANDAN*-treated cells (Fig. 6A), which was comparable to the effect observed due to treatment with standard efflux pump inhibitor carbonyl cyanide 3-chlorophenylhydrazone (CCCP), wherein an increase in EtBr fluorescence of 7.3-fold was observed. A similar trend was also observed when treating the cells with CFS from *L. jensenii.* Additionally, when EtBr efflux was compared between *mutant* strains and the parental strain, the mutant strains accumulated higher levels of EtBr because of the impaired ability to extrude EtBr. CFS treatment also enhanced the EtBr accumulation in the mutant strains by 8.6-fold, which was significantly higher (*p* < 0.001) compared to the parental strain. The combination of (-)-Terpinen-4-ol and itaconic anhydride also exhibited a similar effect, indicating their potential as efflux pump inhibitors (Fig. 6A).

### EtBr efflux

CFS/postbiotics was found to significantly (*P* < 0.001) inhibit the efflux of EtBr in *E. coli* UTI89, as shown by the 2.44-fold increased fluorescence, like that observed with CCCP (2.21-fold). Significant (1.60-fold, *P* < 0.001) inhibition of EtBr efflux was also observed in mutant strains (*∆acrA*,* ∆acrB*,* and ∆tolC*) by CFS/postbiotics, while it was 1.68-fold with CCCP compared to untreated mutant strains (Fig. 6B).

Thus, our findings show that CFS/purified postbiotic metabolites (-)-Terpinen-4-ol and itaconic anhydride exhibits an antipersister effect in conjunction with antimicrobials by enhancing membrane permeability, increasing ROS, and inhibiting efflux. Building on these promising results, we sought to harness the therapeutic potential of these bioactive metabolites by incorporating them into a practical and clinically relevant application. Given their demonstrated efficacy against uropathogenic *E. coli* persisters and their ability to enhance the activity of standard antibiotics, we developed a novel postbiotic-based vaginal wash formulation. This formulation was designed not only to target persistent infections but also to provide broader protection against urinary tract infections (UTIs) through multiple mechanisms of action. To further enhance the formulation’s efficacy, we included tryptamine—another bioactive metabolite derived from Lactobacillus spp. with strong antibiofilm properties proven from our previous study^24^. The combination of (-)-terpinen-4-ol, itaconic anhydride, and tryptamine in a poloxamer 407-based delivery system aimed to create a comprehensive therapeutic solution capable of preventing UTI onset and recurrence, particularly in cases where traditional antibiotics may fail due to resistance or persistence.

### Triple combination of postbiotic metabolites significantly reduces preformed biofilm of *E. coli* UTI89 in vitro

**Fig. 7:**
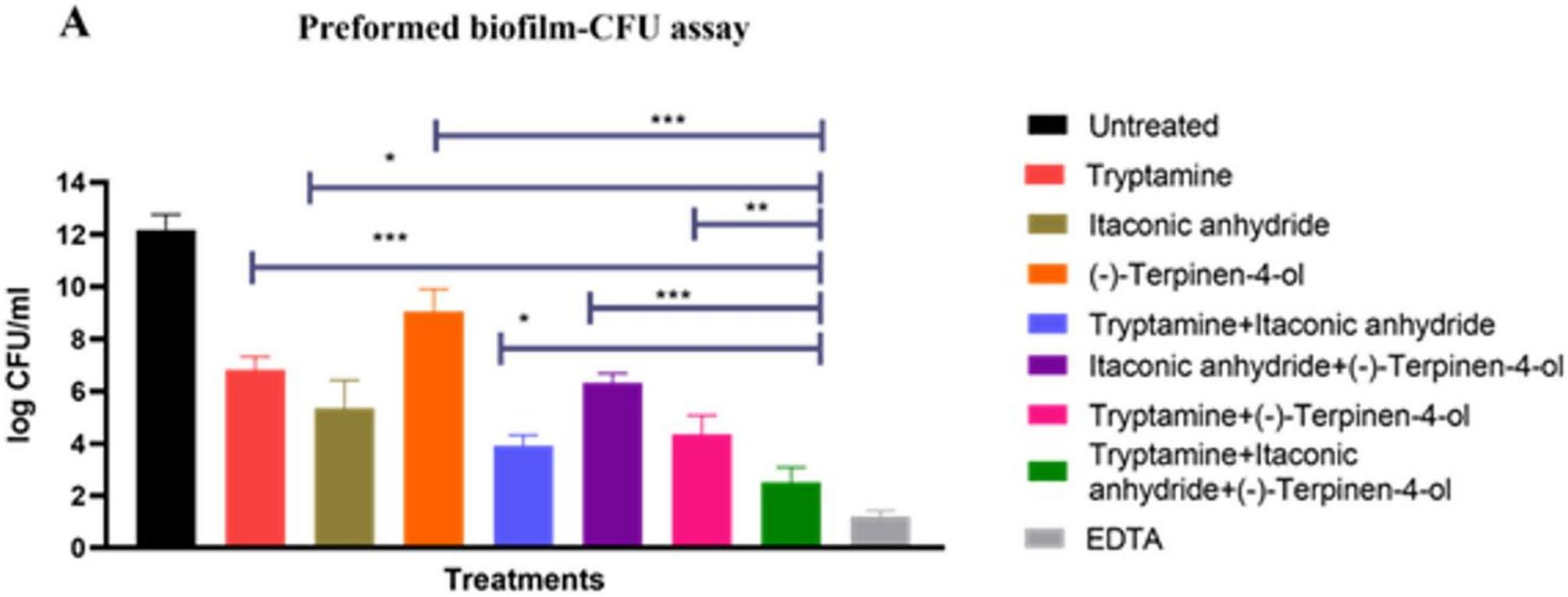
Synergistic Antibiofilm Effect of Postbiotic metabolite combination against Preformed Biofilms of UTI89. Biofilm Reduction in Preformed Biofilms Treated with Individual and Combined Metabolites. A one-tailed t-test was performed to determine the significance. *p < 0.05, **p < 0.01, ***p < 0.001, *n* = 3, EDTA (0.1%) used as a positive control for preformed biofilm.

The efficacy of postbiotic metabolites, tryptamine (4 µg/ml), itaconic anhydride (8 µg/ml), and (-)-terpinen-4-ol (5 µg/ml), was evaluated against preformed biofilms of *E. coli* UTI89 using a colony-forming unit (CFU) assay (Fig. 7A). Treatments included individual metabolites, pairwise combinations, and a triple combination. All treatments significantly reduced biofilm biomass compared to untreated controls, with the triple combination demonstrating the most pronounced reduction, approximating a 10-log decrease in viable biofilm cells.

Additionally, in vitro toxicity studies using Raw macrophages were conducted to assess the safety of these compounds. The MTT assay revealed no significant cytotoxicity at concentrations of 4 µg/ml for tryptamine, 5 µg/ml for (-)-terpinen-4-ol, and 10 µg/ml for itaconic anhydride, indicating that these compounds are safe within the tested concentration range (Fig. S10). These findings suggest that the triple combination of postbiotics not only effectively reduces bacterial burden but also maintains a safety profile, making it a promising candidate for therapeutic applications.

### Development of a customized vaginal wash for enhanced intravaginal delivery of vaginal *Lactobacillus* metabolites

Poloxamer 407 (procured from Sigma-Aldrich), a copolymer known for its unique liquid-to-gel transition upon temperature alteration forms the backbone of the formulation (Fig. S11). Poloxamer 407, characterized by a molecular weight of approximately 12.6 kDa and a hydrophilic nature owing to its polyoxyethylene content (~ 70%), serves as the key component of the formulation. This copolymer’s thermosensitive behaviour, transitioning from a liquid to a gel state in response to temperature changes, enables precise control over gelation kinetics. The addition of tryptamine (4 µg/ml), (-)-terpinen-4-ol (5 µg/ml), and itaconic anhydride (8 µg/ml) further enhances the formulation’s therapeutic efficacy.

The optimal concentration of poloxamer for vaginal drug delivery systems is typically in the range of 18–21% w/w^19^. At an optimised concentration of 18%, Poloxamer 407 confers the formulation with ideal rheological properties, ensuring optimal spreadability and retention within the vaginal cavity. At room temperature (25 °C), the formulation exhibits low viscosity, facilitating ease of application and mucosal coverage (Fig. S11 B). Upon exposure to body temperature (37 °C), the formulation undergoes in situ gelation. Although poloxamer 407–based vaginal gels have been reported to retain integrity for 6–10 h in vivo depending on formulation characteristics, in the present study the gelation was intended to enhance transient mucosal contact during the wash period rather than prolonged vaginal residence^20,21^(Fig. S11A).

The rheological study using Brookfield Viscometer (LVDV-II + Pro, Brookfield Engineering, USA, with spindle no. 64) further demonstrates that the viscosity of the formulation increases significantly with the rise in temperature, indicating its suitability for prolonged retention and enhanced therapeutic efficacy (Fig. S12).

### Biofilm Inhibition and bacterial growth suppression by the customized vaginal wash

**Fig. 8:**
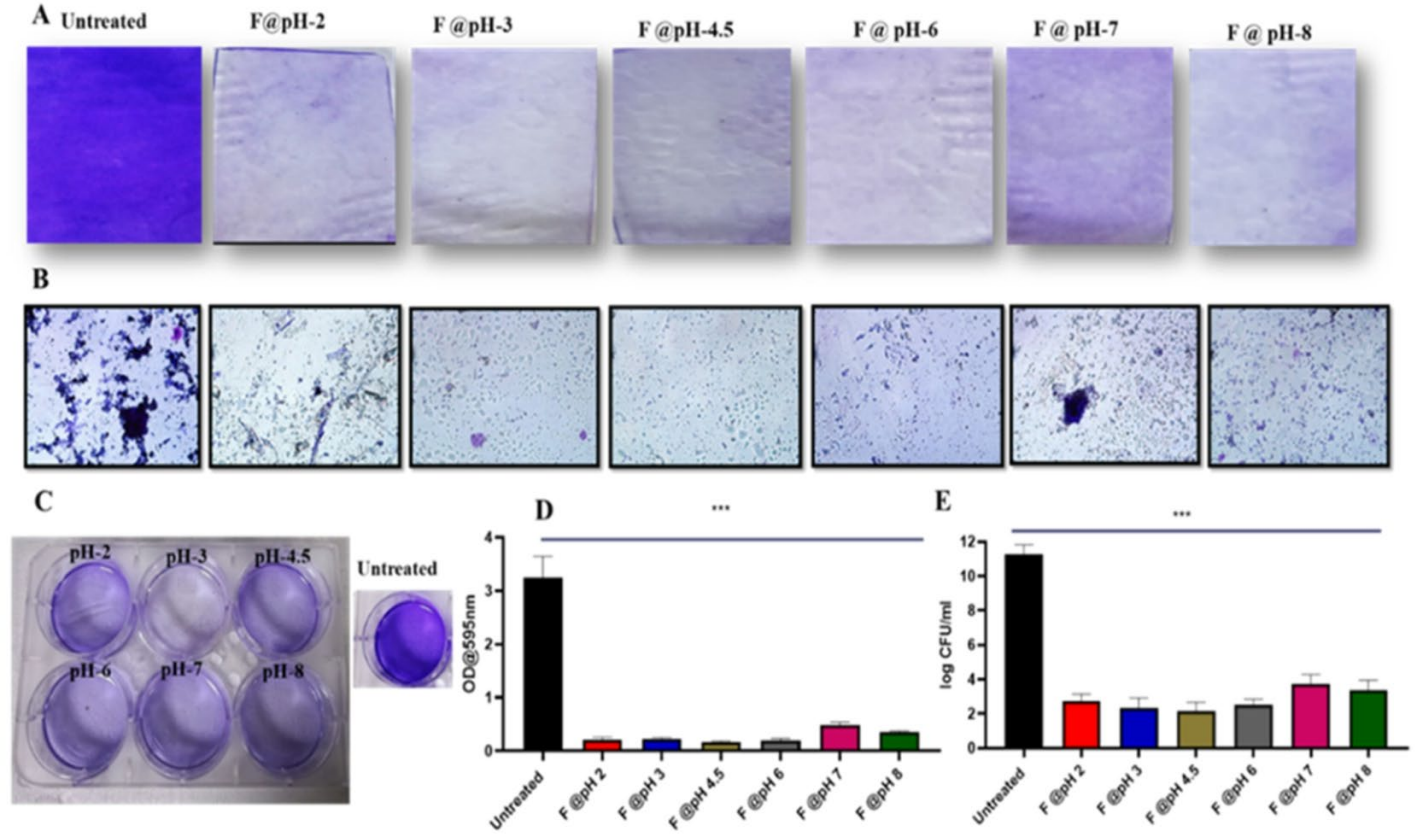
Inhibition of Biofilm formation by the customized vaginal wash formulation **A**) Biofilm formation after treatment with different pH (2, 3, 4.5, 6, 7 and 8) of customized formulation **B**) Microscopic images of biofilm after treatment with different pH of customized formulation **C**) Plate showing Crystal violet assay for biofilm **D**) Quantification of crystal violet assay **E**) Colony forming units. A one-tailed *t-test* was performed to determine the significance. *p < 0.05, **p < 0.01, ***p < 0.001, *n* = 3.

The findings from our study demonstrate the effectiveness of the customized vaginal wash formulation in significantly reducing biofilm formation and bacterial viability across a range of pH levels (Fig. 8A), highlighting its robust inhibitory capabilities. The observed decrease in biofilm density and structural integrity, as evidenced by microscopy (Fig. 8B) and crystal violet assays (Fig. 8C, D), suggests that the formulation effectively disrupts the biofilm matrix, a key factor in preventing persistent bacterial colonization and infection.The 9-log reduction in colony-forming units (CFUs) further confirms the formulation’s capacity to lower bacterial viability within the biofilm, suggesting that it not only inhibits biofilm formation but also actively reduces the existing bacterial population (Fig. 8E).

**Fig. 9:**
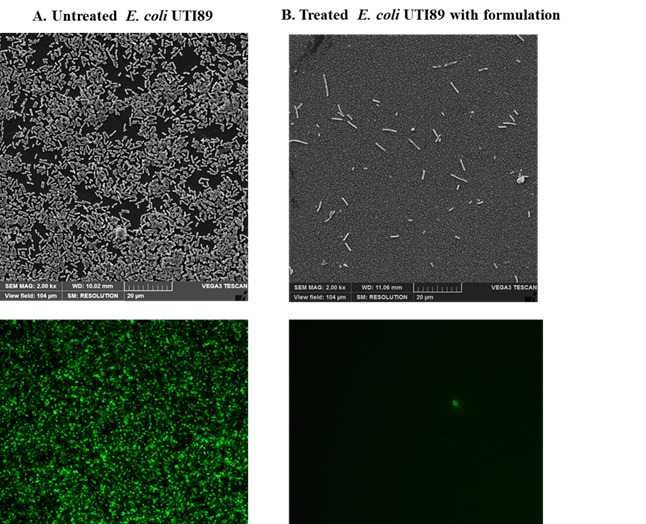
Scanning Electron Microscope and Fluorescent images show the inhibition of bacterial growth after treating with the formulation. (A) Untreated *E. coli* UTI89, (B) Treated with formulation. Scale bar = 20 μm.

Complementing these findings, scanning electron microscopy (SEM) and fluorescence imaging were employed to assess bacterial growth inhibition following treatment with the customized formulation. SEM images revealed a significant reduction in bacterial density, coupled with noticeable morphological abnormalities in the treated groups compared to the untreated control (Fig. 9A). Fluorescence imaging further demonstrated decreased bacterial fluorescence intensity, indicating diminished bacterial viability post-treatment (Fig. 9B).

### Customized vaginal wash formulation retains its stability and biological activity for 3 months at 4 °C

Results revealed that despite the prolonged storage period at 4 °C for 3 months, the customized *Lactobacillus*-derived wash retained its ability to prevent biofilm formation in clinically relevant bacterial strains, including *E. coli* UTI89, *E. coli* CFT073, *E. coli* F11, *Klebsiella pneumoniae*, Methicillin-Resistant *Staphylococcus aureus* (MRSA), and *Pseudomonas aeruginosa*. (Fig. S13 A) and there was ~ 9-log reduction in the growth of all tested bacterial strains (Fig. S13 B). This broad-spectrum efficacy underscores the wash’s potential utility in combating various infections encountered in clinical settings.

### Customized vaginal wash prevents vaginal inflammation in BALB/c mice without affecting body weight

In this study, the efficacy of various vaginal wash formulations was evaluated using a BALB/c mouse model of vaginal infection. The study included five groups (*n* = 6 per group): disease control, placebo, customized vaginal wash, probiotic wash, and commercial wash.

On day 0, a 20 µL suspension containing 10^6^ CFU/mL of *E. coli* UTI89 GFP was instilled into the vaginal cavity of BALB/c mice to induce infection. The inoculation was performed under aseptic conditions to ensure precise bacterial colonization within the vaginal canal. Treatment with the respective vaginal wash formulations began on day 3 post-infection, continuing through day 14 (Fig S14).

**Fig. 10:**
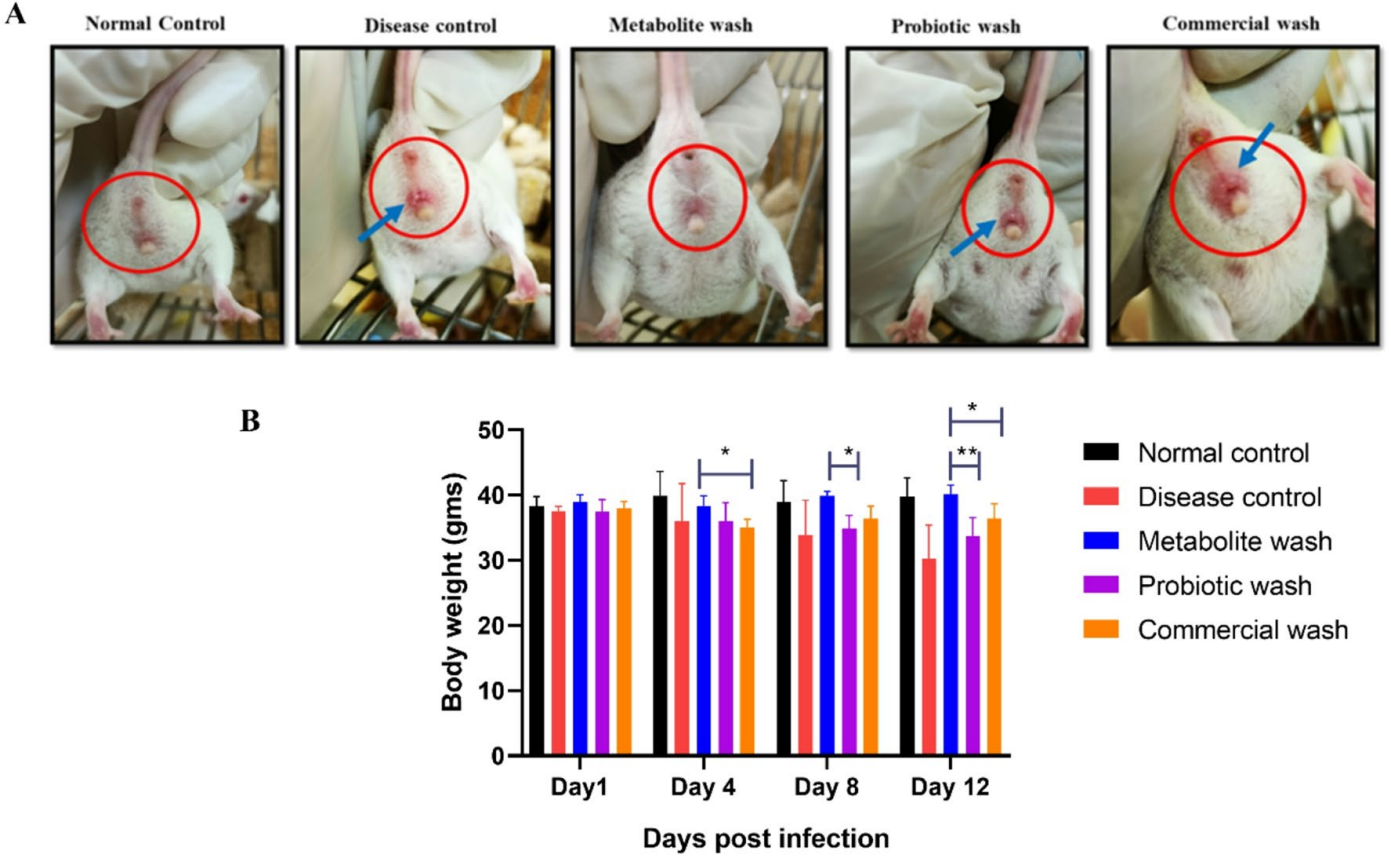
Effect of vaginal wash formulations on inflammation and body weight in BALB/c Mice infected with *E. coli* UTI89 GFP. **A**) Inflammation of the vagina of BALB/c Mice. The blue arrow mark indicates inflammation. **B**) Body weight measurements of BALB/c mice. Data represented as a mean ± standard error of the mean (SEM) (*N* = 6). Statistical significance between indicated groups was determined using one-way ANOVA, *p* < 0.05 (*) and *p* < 0.01 (**).

By day 8, observations revealed significant differences among the treatment groups. The disease control, probiotic wash, and commercial wash groups exhibited pronounced inflammation, including redness, swelling, and the formation of papules. In contrast, the customized vaginal wash-treated group showed significantly reduced inflammation, with vaginal health closely resembling that of the normal control group (Fig. 10A).

Throughout the study, the body weight of the mice was monitored. While the baseline weight ranged between 35 and 40 g, by day 8, the disease control, probiotic wash, and commercial wash groups exhibited a substantial weight loss of approximately 10 g. Notably, mice treated with the customized vaginal wash maintained their baseline weight, indicating better overall health and the efficacy of the customized formulation in managing the infection (Fig. 10B).

### Customized vaginal wash reduced bacterial load in vaginal discharge

**Fig. 11:**
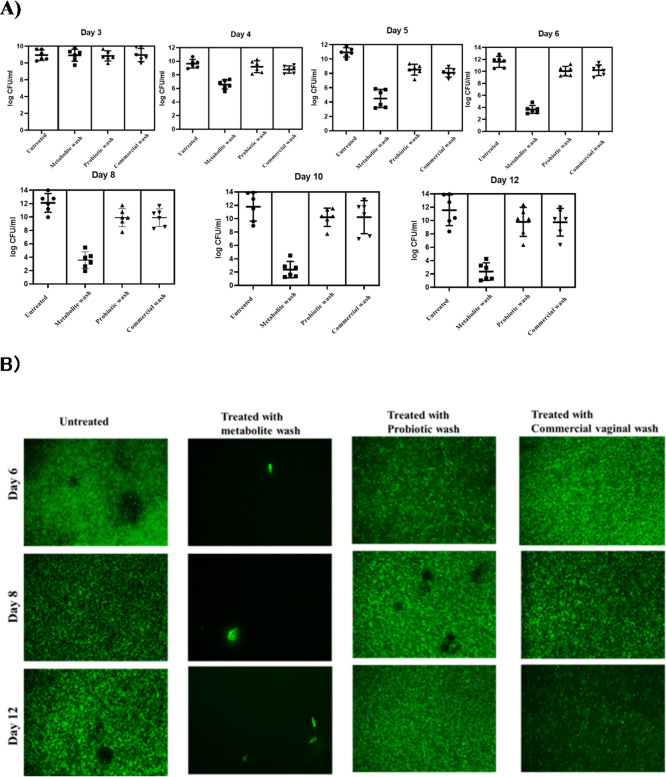
Bacterial loads in the Urine of BALB/c Mice post-infection."0" on the y-axis denotes unsuccessful urine collection. Log 1 on the y-axis represents the baseline (No bacterial count). B) Fluorescent images showing the bacterial load in the urine of BALB/c Mice at Day 8, Day 11, and Day 14 post-infection.

Following formulation treatment, bacterial load in the vaginal discharge of BALB/c mice was quantified (Fig. 11A) using *E. coli *UTI89 GFP for fluorescent imaging. Images at Day 6, Day 8, and Day 12 post-infection showed bacterial colonization dynamics (Fig.11B). The metabolite-based formulation achieved a significant 8-log reduction in bacterial count compared to the untreated control, probiotic formulation, and commercial wash, demonstrating its efficacy in inhibiting bacterial growth and colonization.

### Absence of bacteria in urine/organs after treatment with a customized vaginal wash

We assessed the efficacy of a metabolite-based wash in preventing urinary tract infections (UTIs) and related complications in BALB/c mice. Infection was induced using *E. coli* UTI89 GFP, and bacterial load in urine was quantified at multiple time points post-infection (Fig. 11A). Fluorescent imaging of urine samples collected on Day 8, Day 11, and Day 14 revealed bacterial colonization dynamics within the urinary tract (Fig. 11B). Remarkably, no *E. coli* UTI89 (GFP) was detected in the urine of mice treated with the metabolite wash, indicating a successful prevention of UTI development.

Additionally, we evaluated the bacterial burden in vital organs implicated in UTIs, including the kidney, urinary bladder, and vagina. Mice were sacrificed, and the organs were homogenized to enumerate bacterial load. Mice treated with the metabolite wash exhibited no bacterial load compared to untreated, probiotic, and commercially washed mice. highlighting its protective effects against pyelonephritis and vaginal infections (Fig. S16). The normal creatinine levels were maintained in these mice, further demonstrating the safety and efficacy of the metabolite wash in preventing infection and preserving renal function(Fig S17).

## Materials and methods

### Details of the bacterial strain

Uropathogenic *E. coli* UTI89 (henceforth referred to as *E. coli* UTI89) strain (kindly gifted by Professor Matthew A. Mulvey, University of Utah) and *E. coli* CFT073 were used for all the experiments^22^. The media used for culturing *E. coli* UTI89, and most of the experiments, were performed in Artificial Urine Medium (AUM)^23^ (Yeast Extract Casamino Acids (YESCA) medium (Yeast Extract, 0.5 g/L and Casamino Acids, 10 g/L) The strain was sub cultured, preserved in 20% glycerol , and stored at −80 °C. The compounds, media, and antibiotics were purchased from HI Media Laboratories, India. The antibiotics and compounds were freshly prepared, dissolved in appropriate solvents, and stored at 4 °C.  A standard OD_600_ (~ 0.1) corresponding to ~ 1.0 × 10^7^ CFU/mL was applied throughout the experiment either with or without dilution. All fluorescence based assays were performed using genetix 96 well black microtiter plates (genetixbiotech, Bengaluru, India).

### Isolation, characterization, and Preparation of human vaginal *Lactobacillus* Cell-free supernatant

Vaginal swabs were collected from fifty-four healthy South Indian women aged 18 to 40 years who visited the outpatient clinic with healthy vaginal mucosa at the Obstetrics and Gynaecology Department, Trichy SRM Medical College and Research Centre between June 2022 and July 2022. Before enrolment, all participants provided informed consent. The sample collection procedures strictly followed by the guidelines for human testing established by the institutional and national Human Ethical Committee (IEC No: 127, 696/TSRMMCH&RC/ME-1/2022). Each vaginal swab was obtained from the upper third part of the lateral vaginal wall using HIMEDIA-M HI Culture™ Transport Swabs with Amies Medium without Charcoal. The swabs were then inoculated onto MRS agar (de Man-Rogosa Sharpe Agar, HI media, India) and incubated at 37 °C for 48 h under both microaerobic and anaerobic conditions.

### Identification of bacterial strains

To obtain pure cultures, individual colonies were selected and subcultured. Gram staining and colony morphology were employed to identify *Lactobacillus* at the genus level. To identify the isolated *Lactobacilli* at the species level, the 16S rRNA amplification and sequence analysis methods were employed. Genomic DNA was extracted from Lactobacillus isolates using the QIAamp DNA Micro Kit (Qiagen, Valencia, CA, USA) according to the manufacturer’s instructions and used as a template for PCR amplification. The 16S rRNA gene was sequenced using the forward primer (5′ AGA GTT TGA TCC TGG CTC AG 3′) and the reverse primer (5′ CCC ACT GCT GCC TCC CGT AG 3′), both at a concentration of 0.1 mM. The PCR reaction was conducted in a 50 µl volume containing 5 µl of 10x buffer, 2.5 mM MgCl2, 200 µM of each dNTP, 10 pmol of each primer, 0.5 U Taq polymerase, and 5 µl of *Lactobacillus* DNA, with the remaining volume filled with sterile distilled water. The amplification protocol included an initial denaturation step at 95 °C for 3 min, followed by 30 cycles of denaturation at 95 °C for 1 min, annealing at 48 °C for 1 min, and extension at 72 °C for 2 min, with a final extension step at 72 °C for 10 min. The PCR products were purified using the QIA quick PCR Purification Kit (Qiagen, Valencia, CA, USA) and subsequently analysed using the Applied Biosystems 3730xl DNA Analyzer (Catalogue number: 3730XL) with Dye Terminator Chemistry. Sequences were obtained through a BLAST search, and genus and species were determined using a threshold of 95% sequence similarity. The obtained sequences for the isolated strains were submitted to GenBank (https://www.ncbi.nlm.nih.gov/genbank/) and assigned accession numbers.

Among the isolated *Lactobacillus* strains, *L. fermentum* NANDAN (GenBank accession number: OP648129) and *L. jensenii* (GenBank accession number: OP648111) were selected based on their notable antibacterial and antibiofilm activities. These strains demonstrated high efficacy, prompting us to choose them for further experimental investigations.

### Preparation of cell free supernatant (CFS)

*Lactobacilli* colonies were aseptically inoculated into MRS broth and then incubated at 37 °C for 48 h. The resulting cultures were centrifuged at 10,000×g for 30 min at 4 °C. After centrifugation, the supernatant was adjusted to pH 7.0 with 1 M NaOH and filtered using a 0.22µ disposable syringe filter (HI Media-CC Syringe-Driven filters NYLON-66 Pore size: 0.22µ, India). The filtrate (CFS) was kept at −80 °C for further tests. Filtered CFS was plated on MRS agar plate to ascertain absence microbial cells in the CFS.

### Gas chromatography- mass spectrometry. (GC-MS)

Gas chromatography–mass spectrometry (GC–MS) analysis was performed using a Shimadzu QP2010 SE mass detector to characterize compounds present in column chromatography fractions obtained from the cell-free supernatant (CFS) of *Lactobacillus* spp. The carrier solvent, IJC2 methanol, was selected for its compatibility and efficiency in chromatographic separations. Operating conditions were meticulously optimised to ensure accurate and reliable results. The injection temperature was maintained at 250.0 °C, employing a split injection mode for efficient sample introduction. Within the column oven, a temperature of 50.0 °C facilitated the effective separation of compounds. The system operated at a pressure of 49.7 kilopascals (kPa) with a total flow rate of 54.1 millilitres per minute (mL/min), including a column flow rate of 1 mL/min. Flow control was linear, with a velocity of 36.3 centimetres per second (cm/sec), supplemented by a purge flow of 3.0 mL/min and a split ratio of 8.0 to optimise sample introduction and minimise interference. Mass spectrometry parameters encompassed a scan speed of 1111, covering the mass range from 40 to 450 (mass-to-charge ratio, m/z).

### Determination of minimum inhibitory concentration (MIC)

The minimum inhibitory concentrations (MICs) of itaconic anhydride and (-)-terpinen-4-ol against *E. coli UTI89* were determined using the broth microdilution method in 96 well plate. Stock solutions of itaconic anhydride and (-)-terpinen-4-ol were prepared to cover concentrations ranging from 0 to 256 µg/mL in lysogeny broth (LB) medium. Each concentration was inoculated with 10 µL of *E. coli* UTI89 culture (adjusted to a optical density of  1.0 at 600 nm). After incubation for 24 h at 37 °C, the OD_600_ value in each treated well was determined using a microplate reader (TECAN, Sunrise). The MIC was defined as the lowest concentration of itaconic anhydride/(-)-terpinen-4-ol that visibly inhibited bacterial growth, observed as the absence of turbidity.. All experiments were conducted in triplicate to ensure reliability and reproducibility of results^24^.

### Persister assay of UPEC against *E. coli* UTI89

The UPEC strains were cultured in Artificial Urine Medium (AUM) for 16 h at 37 °C, then diluted 1:100 in fresh medium and incubated until reaching late log phase. In the dose-dependent assay, late log phase cells were exposed to a range of antibiotic concentrations (X MIC) for three hours. In the time-dependent assay, fixed antibiotic concentrations (30X colistin, 40X Ampicillin, 40X Meropenem) were applied, and persister cells were assessed at various time points (3 to 30 h). Post-treatment, cells were harvested by centrifugation at 8000 g for 10 min, washed, and serially diluted in 10 mM phosphate-buffered saline (PBS), pH 7.2. Aliquots of each dilution (10 µL) were spread plated onto YESCA agar to enumerate colony-forming units per ml (CFU/ml). Dilutions yielding 10–100 colonies were selected for further experiments. To evaluate the potential development of resistant cells, treated cells were spread plated on YESCA agar supplemented with antibiotic concentrations (30X colistin, 40X Ampicillin, 40X Meropenem). The concentrations represented as 30X and 40X refer to 30-fold and 40-fold multiples of the minimum inhibitory concentration (MIC), respectively. MIC of Colistin is 2 µg/mL, giving 30X = 60 µg/mL, MIC of Ampicillin is 100 µg/mL, 40X = 400 µg/mL, and Meropenem was used at an MIC of 4 µg/mL, giving 40X = 160 µg/mL. Changes in minimum inhibitory concentrations (MICs) of persister cells obtained after 24 h were also assessed.

Surviving persister cells were transferred to AUM without antibiotics and allowed to grow for 24 h at 37 °C to confirm their nonheritable persister nature. Subsequently, these cells were re-inoculated with 30X colistin, 40X Ampicillin, or 40X Meropenem in AUM. Samples were collected at intervals (every 3 h for 30 h), washed to remove antibiotics, and plated on YESCA agar to detect persister cells. This cycle was repeated three times to assess persistence. The cycle was repeated three times^25^. The results were analysed by using GraphPad Prism8 for Windows.

### Ethidium bromide accumulation assay

For EtBr accumulation assay, 200 µl of late exponential phase cells were transferred to a 96-well black microtiter plate, and CFS or carbonyl cyanide 3-chlorophenylhydrazone, CCCP (8 µl), was then added. After the addition of EtBr (2 µg/ml), EtBr fluorescence was monitored for 60 min at 485 nm and 538 nm for excitation and emission, respectively using Synergy H1 multimode reader (Agilent Technologies, Santa Clara, CA, USA). The growth of the corresponding samples (OD600) was used to normalise the fluorescence^26^. The results were analysed using a t-test using GraphPad Prism8 for Windows.

### Ethidium bromide efflux assay

For the EtBr efflux assay, 800 µl of late exponential phase cells were taken, washed, and re-suspended in PBS before being treated with 10 µl of CCCP (positive control), EtBr (2 mg/ml), and glucose (0.2%). Similar procedures were used on the untreated cell suspension, which served as the control. The samples were harvested, washed twice to get rid of any remnant EtBr, and then re-suspended in PBS after incubationed at 37 °C for 1 h for maximum uptake of EtBr, efflux of EtBr was measured using Synergy H1 multimode reader with excitation and emission wavelengths of 485 nm and 538 nm respectively, the measured EtBr fluorescence was normalised with that of untreated cells^27^. An unpaired t-test was performed using GraphPad Prism 8.

### Reactive oxidative stress assay (ROS assay)

Late exponential phase cells of *E. coli* UTI89 were collected, washed, and resuspended in PBS at a concentration of 10^7^ CFU/ml. Subsequently, the cells were exposed to either 30X colistin (Col), 40X meropenem (Mero), 40X ampicillin (Amp), both individually and in combination with 50 µL of cell-free supernatant or with 1:1 ratio of itaconic anhydride (8 µg/ml) and (-)-terpinen-4-ol (5 µg/ml) respectively for 1 h at 37 °C at 180 rpm. A 10 µM stock solution of 2′,7′-dichlorofluorescin diacetate (DCFH-DA) was added to the cell suspension, and the mixture was incubated at 37 °C for 30 min in the dark. The fluorescence was measured using a Synergy H1 multimode reader with excitation at 485 nm and emission at 538 nm. The fluorescence intensity (FI)/OD600 was calculated to normalize the fluorescence relative to the growth. Untreated cells underwent a similar process and served as the control. For experiments involving reactive oxygen species (ROS) quenching, the cells were treated with antibiotics in the presence of quenchers, specifically 200 mM thiourea. The cells were processed in a similar manner to quantify ROS, as described above^28^. The statistical test used for analysis is unpaired t-test using GraphPad Prism8.

### Membrane permeability assay

To perform the experiments, late exponential phase cells (1.0 mL) were subjected to a pre-treatment step at a temperature of 37 °C for a duration of 1 h. During this pre-treatment, the cells were exposed to 50 µL of CFS or a 1:1 ratio of itaconic anhydride (8 µg/ml) and (-)-terpinen-4-ol (5 µg/ml), and colistin at 30X MIC or Meropenem at 40X MIC. After the pre-treatment period, the cells were harvested, washed, and subsequently re-suspended in 1.0 mL of phosphate-buffered saline (PBS). Fluorescence measurements were performed using a Synergy H1 multimode reader . Each well of the microtiter plate contained 200 µL of the bacterial cell suspension along with 10 µM of NPN (N-Phenyl-1-naphthylamine) as the fluorophore. Fluorescence intensity was immediately measured using an excitation wavelength set at 485 nm and an emission wavelength of 538 nm. To account for variations in cell growth, fluorescence values were normalized with the optical density at 600 nm (OD600) of the corresponding samples. The control group consisted of untreated cells^29^. The results were analysed by using t- test by GraphPad Prism8.

### Scanning electron microscopy

To prepare samples for scanning electron microscopy (SEM), *E. coli* UTI89 cells in the late exponential phase were allowed to form biofilms on 1 cm long catheter pieces. Subsequently, the biofilms were exposed to 50 µL of cell-free supernatant (CFS) or a combination of 1:1 ratio of itaconic anhydride (8 µg/ml) and (-)-terpinen-4-ol (5 µg/ml), 30X MIC of Colistin, and 40X MIC of Meropenem for 1 h and 3 h. The samples were then centrifuged at 1400×g for 5 min. Following centrifugation, the cells were washed twice with pH 7.4 phosphate-buffered saline (PBS) and fixed overnight at 4 °C using a 2.5% glutaraldehyde solution. After fixation, the cells were washed thrice with pH 7.4 PBS and subjected to dehydration using a series of graded ethanol concentrations (ranging from 30% to 100%). Both the treated samples and the untreated control cells underwent the same sample preparation procedure. The dehydrated samples were mounted onto aluminium stubs, sputter-coated with a layer of gold-palladium, and desiccated. The resulting samples were then examined using a digital field emission scanning electron microscope (TESCAN VEGA 3, BRNO, CZECH REPUBLIC), and electron micrographs were captured for analysis.

### In vitro cell line toxicity study

 Cytotoxicity of the postbiotic metabolites was evaluated using RAW (264.7) macrophages by the MTT assay. Cells were cultured in DMEM medium supplemented with 10% fetal bovine serum (FBS). RAW macrophages were seeded into 96-well plates and allowed to reach 70–80% confluence before treatment. Cells were exposed to the test compounds at the concentrations used in functional assays for 24 h, followed by incubation with MTT (0.5 mg/mL) for 2 h. Formazan crystals were dissolved using DMSO, and absorbance was measured at 595 nm to assess cell viability relative to untreated controls^30^. to assess cell viability.

### Biofilm formation

A 96-well microtiter plate was used, with 200 µL of test medium and 20 µL of a 48-hour LB broth culture of *E. coli *UTI89/ other tested microbe as the inoculum. After incubating at 37 °C for 48 h under static conditions, unbound cells were washed with sterile PBS. Biofilm-bound cells were stained with 1% Crystal Violet for 15 min, rinsed with distilled water, and the bound dye was extracted with 70% ethanol. Absorbance was measured at 595 nm. Viable cells were determined by scraping the wells, performing a 10-fold dilution, and spread plating to enumerate colony-forming units (CFU)^22^. Treatments were administered with a triple combination of postbiotics at different pH levels.

### Rheological analysis

To assess the viscosity of the formulation at varying temperatures, a Brookfield Viscometer (LVDV-II + Pro) with Spindle No. 64 was used. The formulation was equilibrated at the initial temperature, and its viscosity was measured. The sample was then subjected to incremental temperature increases, with viscosity readings taken at each step after allowing the formulation to stabilize. The results, indicating viscosity changes with temperature, demonstrate the formulation’s suitability for prolonged retention and enhanced therapeutic efficacy, as a significant increase in viscosity with rising temperature suggests better performance under therapeutic conditions.

### In vivo infection study using BALB/c mice

Animal study Institutional Animal Ethical Committee of SASTRA Deemed to be University (Approval number: (740/SASTRA/IAEC/RPP)). The animal experiments reported are found to be in accordance with ARRIVE guidelines.

The in vivo study was organized into five groups (*n* = 6 per group): (i) normal control (uninfected, untreated) to establish baseline parameters; (ii) disease control (*E. coli* UTI89–infected, untreated) to assess infection progression; (iii) placebo group (infected, treated with formulation base alone) to evaluate vehicle effects; (iv) customized vaginal wash group (infected, treated with postbiotic formulation) to assess therapeutic efficacy; and (v) commercial wash group (infected, treated with a reference vaginal wash) for comparative evaluation.A pilot study was conducted before the main study to optimise the experimental parameters and determine the appropriate study duration. The main study, spanning 14 days, involved vaginal induction of *E. coli* UTI89 GFP in BALB/c mice by administering 20 µL of a bacterial suspension (10^6 CFU/mL) directly into the vaginal canal using a sterile pipette under aseptic conditions, ensuring precise and uniform colonisation. Following induction, the mice were monitored for signs of infection, such as inflammation and vaginal discharge, with body weight recorded regularly. Treatment with the vaginal wash formulations were applied meticulously. Urine samples were collected on days 8, 11, and 14, and Vaginal discharge samples were collected on days 3, 4, 5, 6, 8, 10, and 12 by gently washing the vaginal opening with a sterile pipette containing a small volume of sterile phosphate-buffered saline (PBS). The recovered lavage fluid was collected aseptically and used for bacterial enumeration and fluorescence analysis. Care was taken to ensure minimal discomfort and consistent sample collection across all groups. Blood samples were collected for serum creatinine assays (AGAPPE Diagnostics Ltd, Mumbai, India) to assess renal function. Mice were sacrificed, and their organs were homogenised to enumerate bacterial load. Detailed schematics of the animal study are provided in Fig. S15.

For the preparation of the probiotic wash, 0.01 M lactic acid/sodium lactate buffer (pH 3.2) was cooled to 4 °C. To this buffer, 18% Poloxamer and freeze-dried bacterial powder were slowly added with continuous agitation. *Lactobacillus jensenii* was introduced at a concentration of 11 log CFU/mL, and the mixture was left at 4 °C until a clear solution was obtained, achieving a final pH of 3.8.

The Poloxamer-407 formulation containing postbiotics was prepared by combining 18% Poloxamer with tryptamine (4 µg/mL), (-)-Terpinen-4-ol (5 µg/mL), and itaconic anhydride (8 µg/mL). For the commercial wash, 1 mL of the commercial vaginal wash was diluted with 99 mL of water.

### Statistical analysis

Statistical analyses were performed using GraphPad Prism (version 8). Comparisons between two groups were conducted using an unpaired Student’s t-test, while comparisons among multiple groups were analysed using one-way ANOVA, followed by Tukey’s post hoc test where applicable. A one-tailed t-test was used for biofilm inhibition assays, as specified. Statistical significance was defined as *p* ≤ 0.05, with levels of significance indicated as *p* ≤ 0.05 (*), *p* ≤ 0.01 (**), and *p* ≤ 0.001 (***).

## Discussion

In the present study, we identified and characterized bioactive metabolites present in the cell-free supernatant (CFS) of vaginal *Lactobacillus spp*. that contribute to inhibition of biofilm matrix production and eradication of persister cells in uropathogenic *Escherichia coli* (UPEC). Among the identified metabolites, tryptamine, a biogenic amine, was shown earlier by us^11^ to inhibit biofilm matrix formation in UPEC (Fig S1). Indole alkaloids such as tryptamine are known for diverse biological activities, including anticancer, antimalarial and antimicrobial effects^31^. Several bacterial genera , including *Lactobacillus*, *Staphylococcus*, and other Gram-positive organisms, produce tryptamine via aromatic amino acid decarboxylase activity under both aerobic and anaerobic conditions^32^. Tryptamine has demonstrated inducible effects on serotonin secretion^33^ and shows inhibitory effects on tryptophanyl-tRNA synthetase (TrpRS), the inhibition of TrpRS subsequently disrupts protein biosynthesis, resulting in cytotoxic effects on microbial cells^34^. Itaconic anhydride, the cyclic form of itaconic acid & (−)-terpinen-4-ol represents the other key metabolites identified in this study [Fig. 3]. Itaconic acid is known for its antimicrobial and anti-inflammatory properties, primarily through inhibition of isocitrate lyase in the glyoxylate shunt, a pathway critical for bacterial survival under nutrient-limited conditions^35^. We had previously shown that the vaginal *Lactobacilli *derived Terpinen-4-ol displayed potent efflux inhibitory effect against UPEC [Fig S2]^12^. Terpinen-4-ol, a naturally occurring monoterpene, is well recognized for its potent antibacterial and antibiofilm activity^36^, Its mechanism of action involves disruption of bacterial membrane integrity, increased permeability, and leakage of intracellular contents, leading to impaired cellular function^37,38^.In the present study, the combination of itaconic anhydride and (−)-terpinen-4-ol exhibited synergistic activity against *E. coli* persister cells [Fig. 3], highlighting their complementary modes of action.

Persistent bacterial infections remain a major clinical challenge, particularly in conditions such as tuberculosis, lyme disease, cystic fibrosis, and recurrent urinary tract infections (UTIs). These infections are often driven by persister cells—phenotypic variants that exhibit high tolerance to antibiotics and environmental stresses^13^. Persisters are difficult to eradicate due to their dormant state, low metabolic activity, and similarity to viable but non-culturable cells^39^. In UPEC, persister cells contribute significantly to infection relapse and chronicity, while excessive antibiotic use accelerates resistance development. Therefore, developing strategies to eradicate persister cells is urgently needed. Anti-persister approaches broadly include direct killing of dormant cells, resensitization to antibiotics, or inhibition of persister formation^40^. However, many existing strategies require metabolic stimulation or membrane potential restoration, which are difficult to achieve in vivo^41^.Traditional antibiotic discovery platforms primarily identify growth-inhibitory compounds and are often ineffective against persisters^42^. These limitations underscore the need for alternative, non-antibiotic strategies.

Probiotics and their derivatives have gained attention as potential interventions for recurrent UTIs^43^. Vaginal *Lactobacillus *species exhibit strain-specific antimicrobial activity through production of metabolites, biofilm disruption, immune modulation, and competitive exclusion^44^. Several *Lactobacillus* species, including *L. fermentum*, *L. pentosus*, and *L. acidophilus* have demonstrated efficacy against biofilms and persister cells^45^. *E. coli* Nissle 1917 has been shown to reduce persister survival through contact-dependent mechanisms^46^. In this study, we demonstrate for the first time that postbiotics derived from vaginal *Lactobacillus*, specifically itaconic anhydride and (−)-terpinen-4-ol in combination, effectively curtails *E. coli* UTI89 persister cells (Fig. 3). Earlier studies have also shown that Itaconic anhydride elevates ROS levels in host immune cells and suppress bacterial survival pathways^47^, while terpinen-4-ol disrupts membrane integrity and interferes with essential cellular processes^48^. These complementary mechanisms underpin the observed synergistic activity.

Exposure of *E. coli* UTI89 to high concentrations of colistin, ampicillin, and meropenem resulted in persister cell formation (Fig. 1), consistent with earlier reports of antibiotic tolerance in UPEC under urine-mimicking conditions^49^. The observed prooxidant effect of itaconic anhydride (Fig. 4), coupled with the membrane-disrupting action of terpinen-4-ol (Fig. 5), significantly reduced persister survival. The increased survival of persister cells in the presence of the ROS quencher thiourea further confirms the critical role of ROS in this process (Fig. S8). Scanning electron microscopy (Fig. S9 E& F) revealed pronounced morphological damage in persister cells treated with the postbiotic combination, including membrane disruption and surface irregularities, consistent with previous reports on terpinen-4-ol and postbiotic-induced cellular damage^50^. Ethidium bromide accumulation assays demonstrated that itaconic anhydride and terpinen-4-ol inhibits efflux pump activity, further enhancing intracellular antimicrobial accumulation and contributing to persister eradication (Fig. 6). These findings indicate that postbiotic metabolites from vaginal *Lactobacillus* spp. act synergistically by enhancing membrane permeability, elevating ROS levels, and inhibiting efflux systems, thereby effectively targeting UPEC persister cells.

**Fig. 12:**
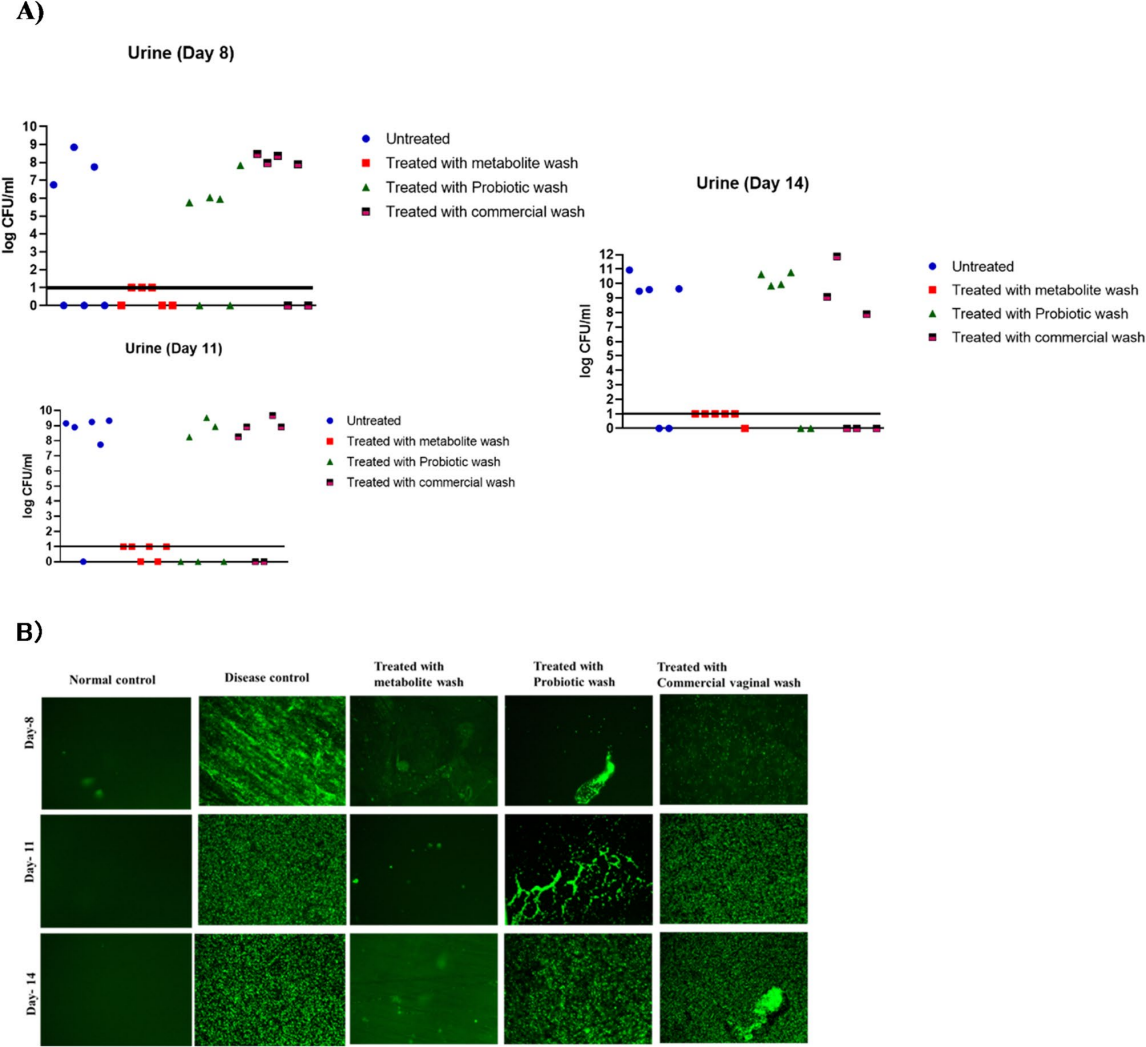
Bacterial loads in vaginal discharge of BALB/c Mice post-infection. **A**) Quantifications were performed in triplicate and represented as a mean ± standard error of the mean (SEM) (*N* = 6). **B**) Fluorescent images showing the bacterial load in the vaginal discharge of BALB/c Mice at Day 4, Day 8, and Day 12 post-infection. (*N* = 6).

The in vivo findings suggest that the customised vaginal wash offers a promising therapeutic strategy for managing bacterial infections and preventing UTIs in addition to the observed reduction in bacterial burden in vaginal discharge (Fig. 12), Urine (Fig. 11), in various organs (Fig S16) and the pronounced reduction in inflammation (Fig. 10), the formulation itself contributes substantially to therapeutic performance. Poloxamer-based systems, particularly poloxamer 407, were selected due to its well-documented biocompatibility, thermoresponsive gelation, and mucoadhesive properties, which enhances local residence time and sustained release at mucosal surfaces^51^. Previous studies have demonstrated that poloxamer formulations are well tolerated in vaginal and urogenital applications and can improve antimicrobial efficacy by prolonging contact with the epithelium while minimizing systemic exposure^52^. Relative to the other non‑antibiotic UTI interventions tested in murine models, including prophylactic herbal regimens^53^, Tailin Fang II decoction in the UPEC CFT073 infection model^54,^ cranberry‑derived anti‑adhesion/anti‑filamentation bioactive^55^, and probiotic‑based strategies delivered orally as mixed viable/heat‑killed composites^56^, our approach uniquely combines a locally acting, formulation-driven strategy with targeted antimicrobial activity. While herbal and probiotic interventions primarily modulate host immunity or bacterial adhesion over prolonged administration periods, the present formulation provides a rapid, localised effect at the vaginal–urethral interface, which is critical for early intervention and recurrence prevention. This formulation-based strategy therefore complements existing non-antibiotic approaches and highlights the translational potential of vaginal delivery systems in UTI management.

## Conclusion

Our study presents a novel and promising approach for the prevention and management of urinary tract infections (UTIs) by utilising a customised *Lactobacillus* metabolite wash. This formulation demonstrates significant efficacy in reducing bacterial colonisation and biofilm formation, which are critical factors in the persistence and recurrence of UTIs. The ability of this metabolite-based wash to effectively prevent UTIs, without relying on antibiotics, positions it as a valuable alternative to conventional treatments, particularly in the context of rising antibiotic resistance and the disruption of natural microbiota associated with traditional antibiotic therapies. The customised *Lactobacillus* metabolite wash not only showed a marked reduction in bacterial load in key organs such as the urine, kidneys, urinary bladder, and vagina, but also maintained the overall health and body weight of the treated mice. This indicates its potential to reduce the risk of pyelonephritis and secondary infections, further underscoring its therapeutic value. Moreover, our study is the first to report the synergistic action of Itaconic anhydride and (-)-Terpinen-4-ol, metabolites derived from Lactobacillus, in exhibiting potent antipersister activity. These metabolites act as prooxidants, effectively inhibiting the formation of persister cells, which are often responsible for chronic infections and post-treatment relapses. By inhibiting efflux pumps, these compounds enhance the intracellular concentration of antibiotics such as colistin and meropenem, thereby boosting their efficacy against persistent bacterial infections. These findings suggest that targeting bacterial persister cells with *Lactobacillu*sderived metabolites, in combination with antibiotics, could offer a powerful strategy for treating recurrent UTIs, particularly those mediated by *E. coli* UTI89. The customised metabolite wash, with its ease of administration, minimal side effects, and compatibility with regular hygiene routines, represents a significant advancement in UTI prevention. It holds the potential to enhance the quality of life for women by promoting vaginal health and providing a sustainable, cost-effective alternative to traditional UTI treatments.

## Supplementary Information

Below is the link to the electronic supplementary material.

Supplementary Material 1

## Data Availability

The 16srRNA sequence datasets generated and/or analysed during the current study are available in the NCBI GenBank repository, The organism’s name and the accession number are as follows: Lactobacillus jensenii strain NANDAN (Accession No: OP648111), Limosilactobacillus fermentum strain NANDAN (Accession No: OP648129). All relevant data has been comprehensively presented in the main text and supplementary materials. Raw data is available upon request at sai@scbt.sastra.edu.
