# Supplementary material for: Synergistic antipersister, efflux inhibitory & antibiofilm activities of vaginal Lactobacillus-derived postbiotics against UPEC: toward a novel therapeutic for utis
Source: Sci Rep. 2026 Jan 10;16:5005. doi: 10.1038/s41598-026-35736-7 (PMC12876057; doi:10.1038/s41598-026-35736-7)
Supplement: Supplementary file 1 — Supplementary Material 1 [file 41598_2026_35736_MOESM1_ESM.docx]

**SUPPLEMENTARY DATA**

**Synergistic Antipersister, Efflux Inhibitory & Antibiofilm Activities of Vaginal Lactobacillus-Derived Postbiotics Against UPEC: Toward a Novel Therapeutic for UTIs**

**Veena G Nair ^1^, David Raj Chellappan^2^, Ramya Devi Durai^3^, Rajesh Y.B.R.D ^4^, Dhiviya Narbhavi^5^, Anupriya A^6^, Prabhusaran N^7^, Saisubramanian Nagarajan ^1*^**

*** Corresponding Author: Dr. Sai Subramanian N, Antimicrobial Resistance Lab, Centre for Research in Infectious diseases, School of Chemical and Biotechnology, SASTRA Deemed to be University, Thanjavur 613401, Tamil Nadu, India. Phone: +91 4362 2641011**

**email: sai@scbt.sastra.edu**

**^1^ Antimicrobial Resistance Lab, Centre for Research in Infectious diseases, School of Chemical and Biotechnology, SASTRA Deemed to be University, Thanjavur 613401, Tamil Nadu, India.**

**^2^ Central Animal House Facility, SASTRA Deemed to be University, Thanjavur, 613401, India**

**^3^Pharmaceutical Technology Laboratory, School of Chemical and Biotechnology, SASTRA Deemed to be University, Thanjavur, 613401, India**

**^4^Department of Chemistry, School of Chemical and Biotechnology, SASTRA Deemed University, Thanjavur – 613 401, Tamil Nadu, India.**

**^5^ Assistant Professor, Department of Obstetrics and Gynaecology, TSRMMCH&RC, Tiruchirappalli, Tamil Nadu, India**

**^6^Associate Professor, Department of Microbiology, TSRMMCH&RC, Tiruchirappalli, Tamil Nadu, India**

**^7^ Research Faculty, Institutional Research Board TSRMMCH&RC, Tiruchirappalli, Tamil Nadu, India**


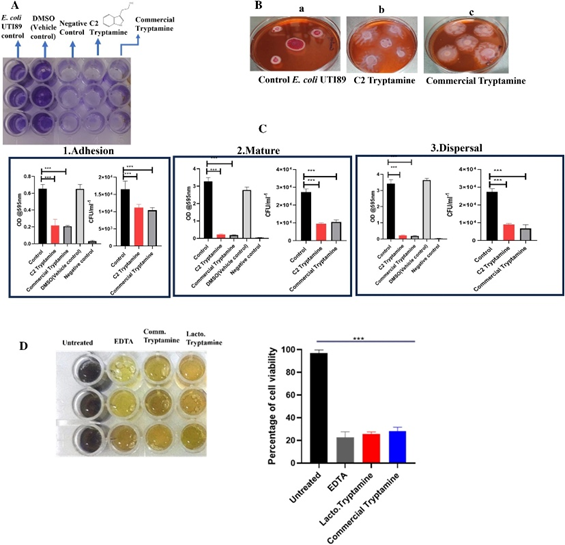


**Fig. S1.** Tryptamine inhibits matrix production and biofilm formation in UPEC. (A) Crystal violet assay showing reduced biomass. (B) Colony morphology shifted from RDAR to SAW, indicating matrix loss. (C) Inhibition across adhesion, maturation, and dispersal stages. (D) Reduced biofilm cell viability in the MTT assay. Reproduced with permission from Nair *et al*., 2024 *(Scientific Reports https://doi.org/10.1038/s41598-024-65780-0)*


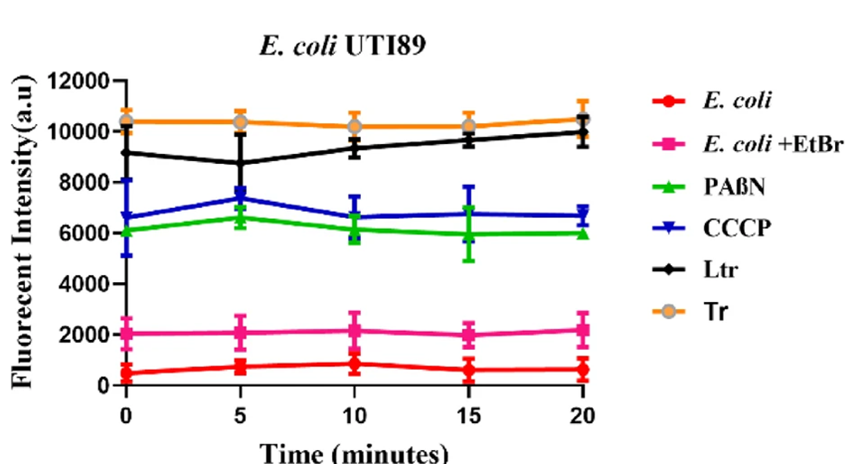


**Fig. S2.** (-)-Terpinen-4-ol from Lactobacillus jensenii (Ltr) and commercial (-)-Terpinen-4-ol (Tr) inhibited EtBr efflux in *E. coli* UTI89. Efflux inhibition was comparable to standard controls (PABN and CCCP). Reproduced with permission from Nair *et al*., 2025 (*Scientific Reports* https://doi.org/10.1038/s41598-025-17404-4)

e

f

d


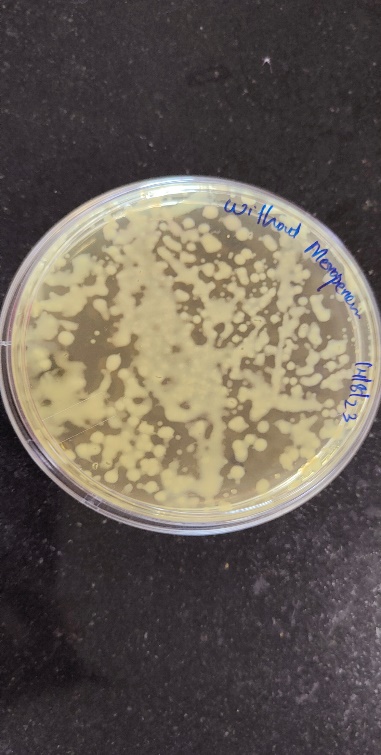

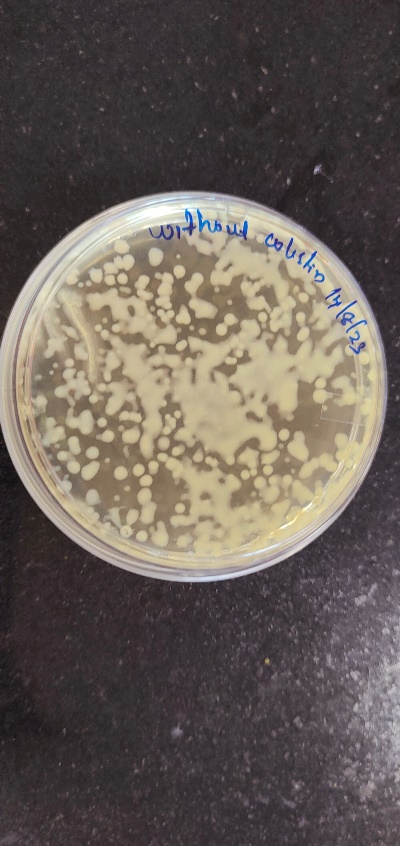

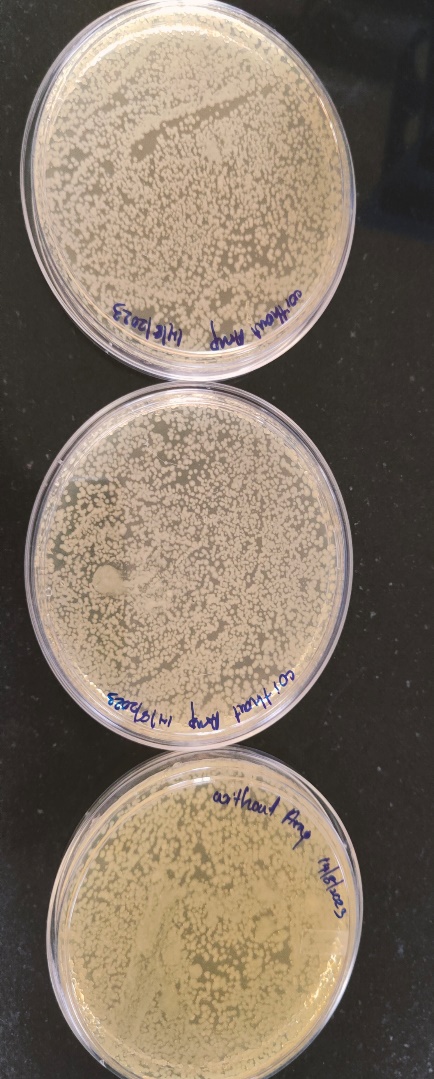


b

c

a


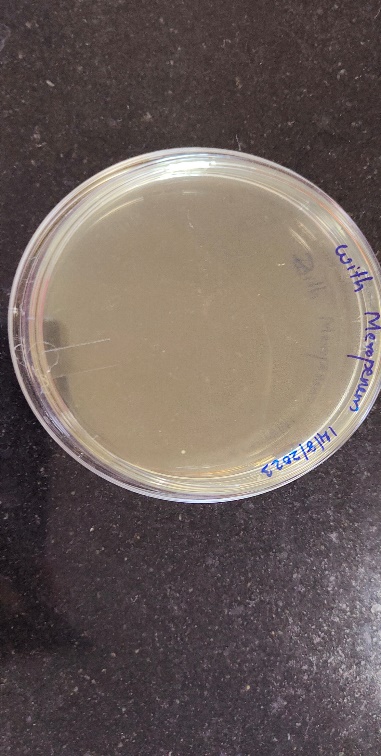

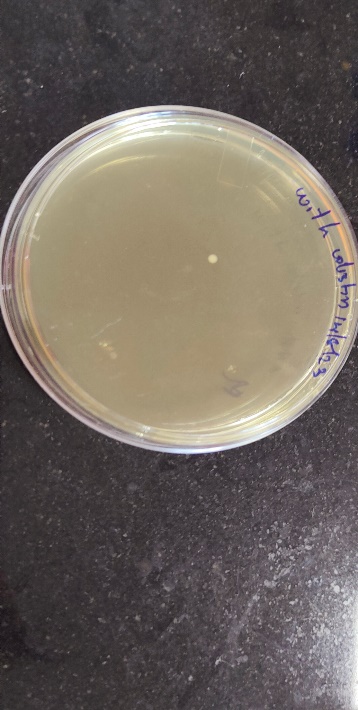

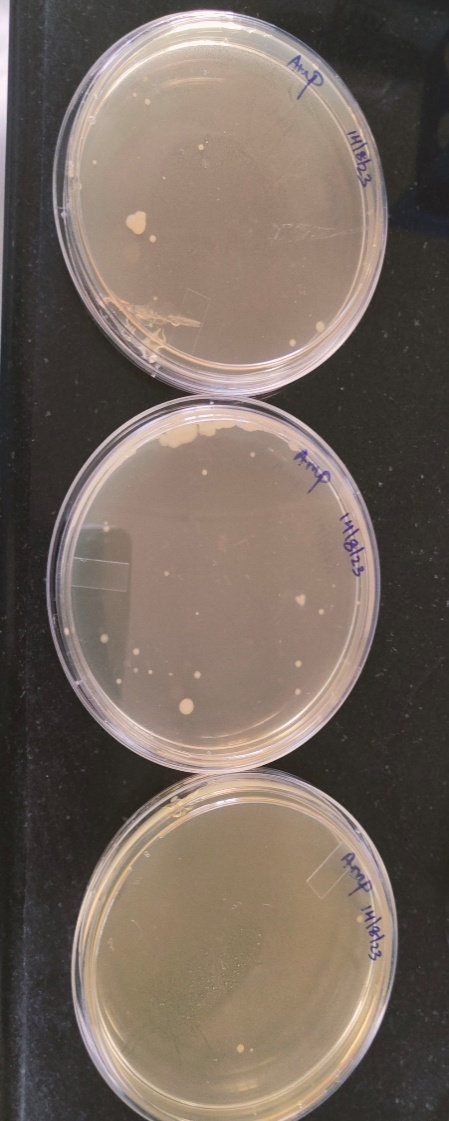


**Fig. S3**: Plates demonstrating that the persister cells were not resistant to antibiotics, a) With Meropenem, b) With colistin, c) With Ampicillin, d)Without Meropenem, e) Without colistin, f) Without Ampicillin. (N=2)

**F**


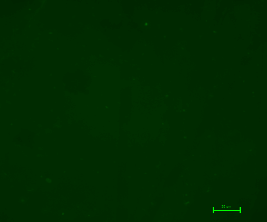


**A. Ampicillin**


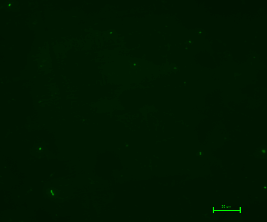


**B. Meropenem**


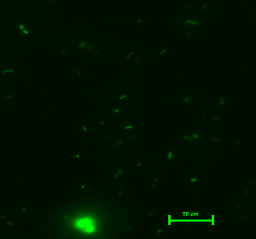


**C. Colistin**


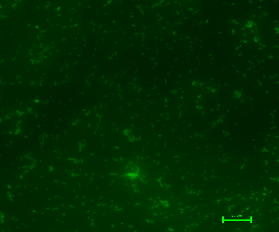


**D**. CFS of *L. fermentum*

treated

**E**. CFS of *L. jensenii* treated.


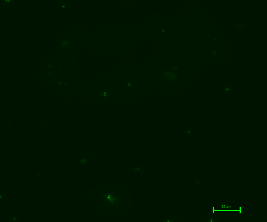

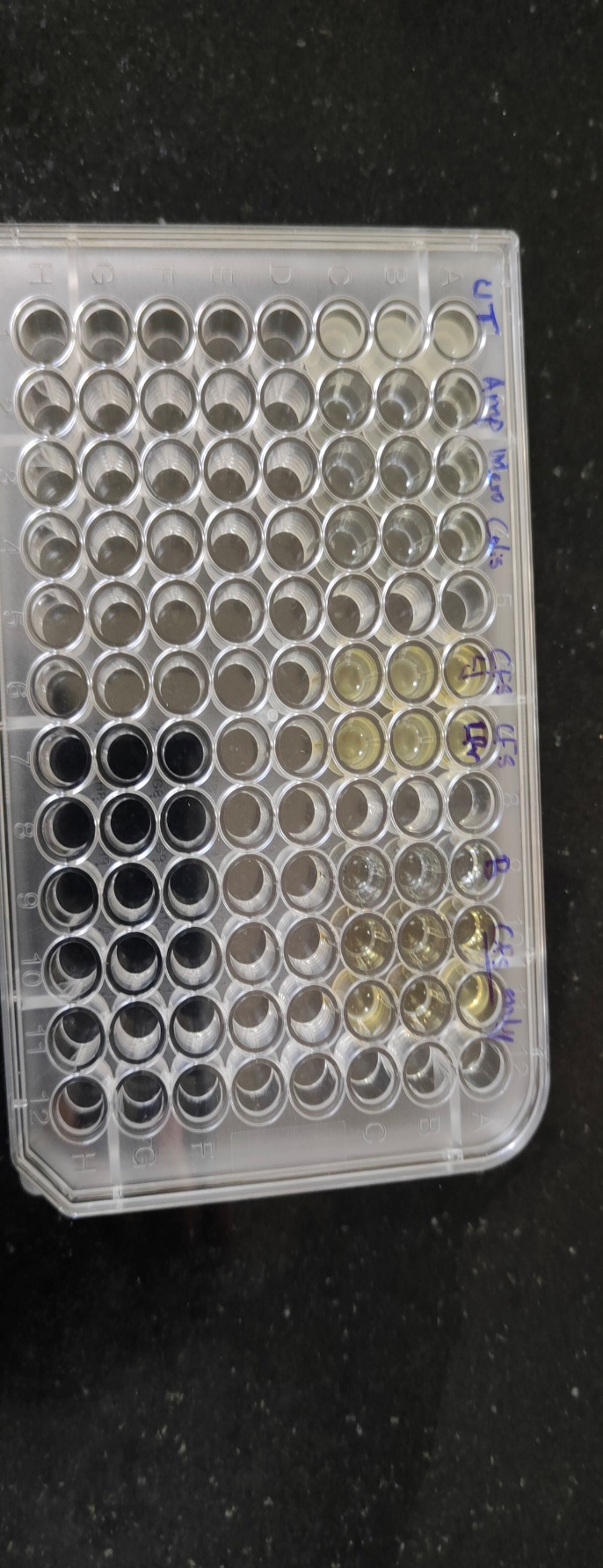


**Fig. S4:** Fluorescent Microscopic images showing that formed persister cells were not resistant to antibiotics and it grows on cell free supernatant. **A, B, C** GFP tagged *E. coli* UTI89 persister cells failed to grow in the MIC concentration of Ampicillin, Meropenem and Colistin. **D, E**. GFP tagged Persister cells had grown in the presence of Cell free supernatant of *L. fermentum* and *L. jensenii* respectively**. F.** There was no growth observed in the presence of antibiotics (marked in Red ), and there was growth in the presence of CFS (marked in black)


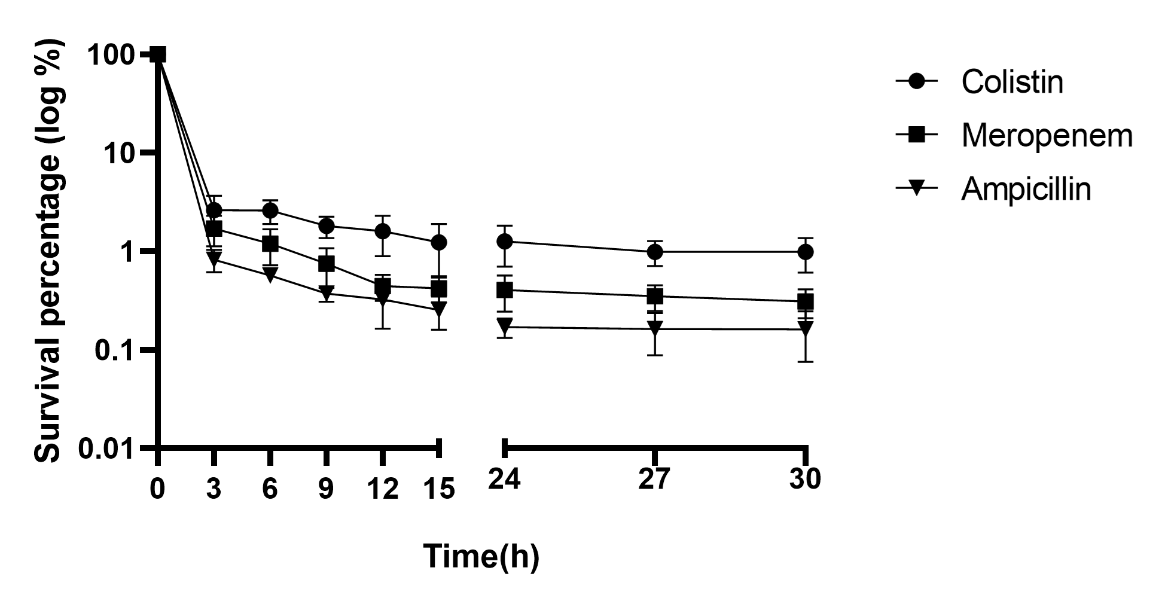


**Fig. S5**: **Time dependent persister assay:** *E. coli* UTI89 treated with Colistin (30X MIC), Ampicillin (40X MIC) and Meropenem (40X MIC) respectively at different time points. Untreated cells (100% survival) taken as the control were 2×10^9^, 3.9×10^9,^ and 2.6×10^9^ CFU/ml, respectively. Three independent experiments are represented by the data. Error Bars represent the mean ± SD.

**A**

**B**

**C**

**Fig. S6**: **Persister cell test for non-heritability.** The cells were then subjected to >30 X MIC levels of antibiotics for 3 hours over three growth phases, followed by treatment with antibiotics A) Colistin (30X MIC), B) Ampicillin (40X MIC), and C) Meropenem (40X MIC). The data is representative of three independent experiments. Bars represent the mean ± SD.

**A**

**B**


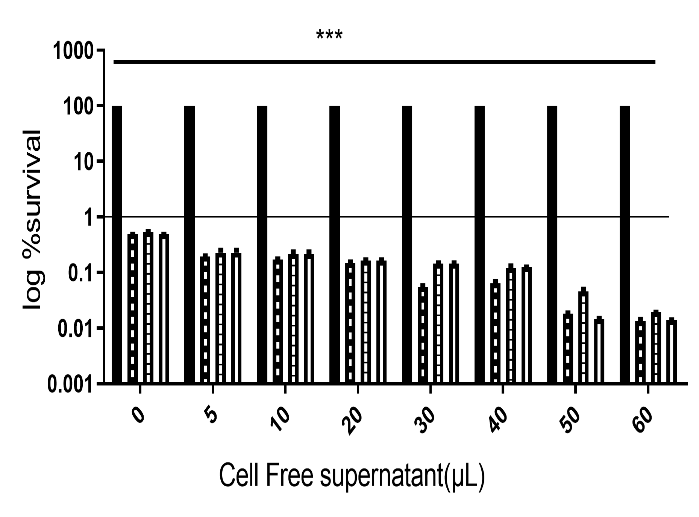

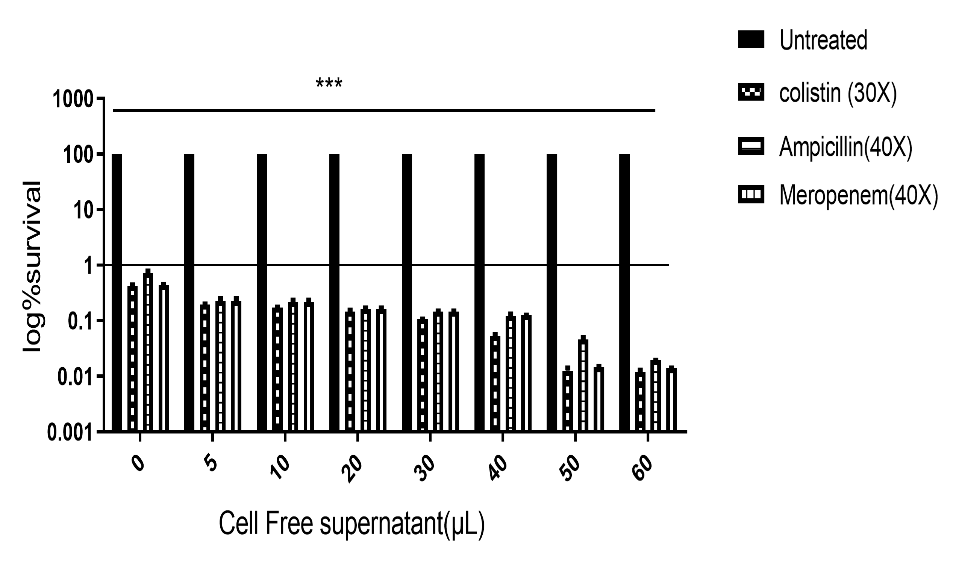


**Fig. S7**: Effect of CFS of *L. fermentum* NANDAN and *L. jensenii* on persister cell formation *E. coli UTI89* cells treated for 12 hours with cell-free supernatant (CFS) in combination with 10X colistin, 40X meropenem, and 40X ampicillin, at varying concentrations of 0-60 µL of CFS.**A**) *L. fermentum NANDAN,* **B**) *L. jensenii.* The data is representative of three independent experiments. Bars represent the mean±SD. ***p≤0.001.


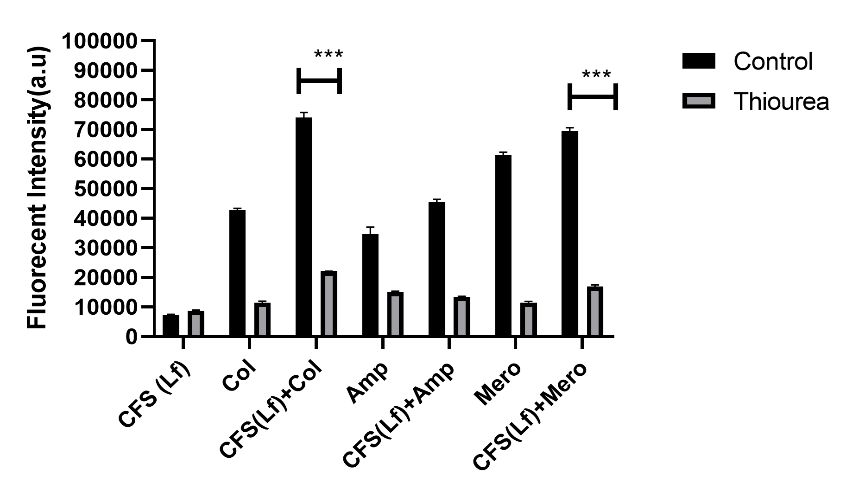

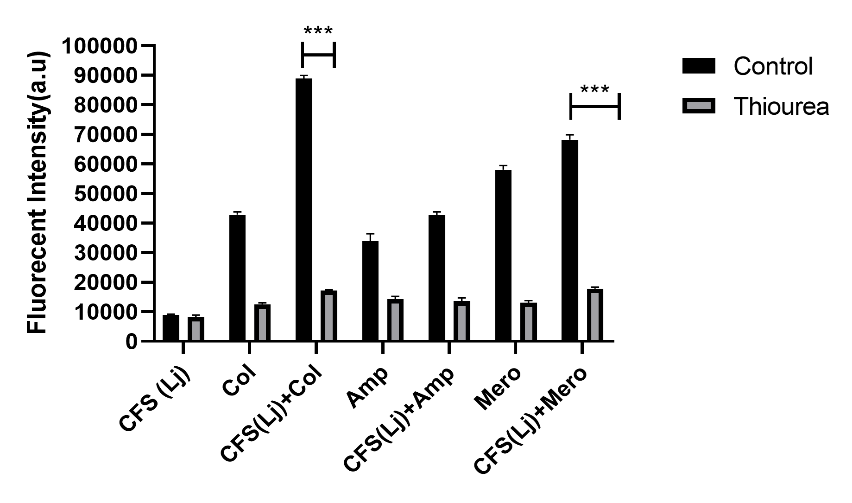


**A)**

**B)**

**C)**


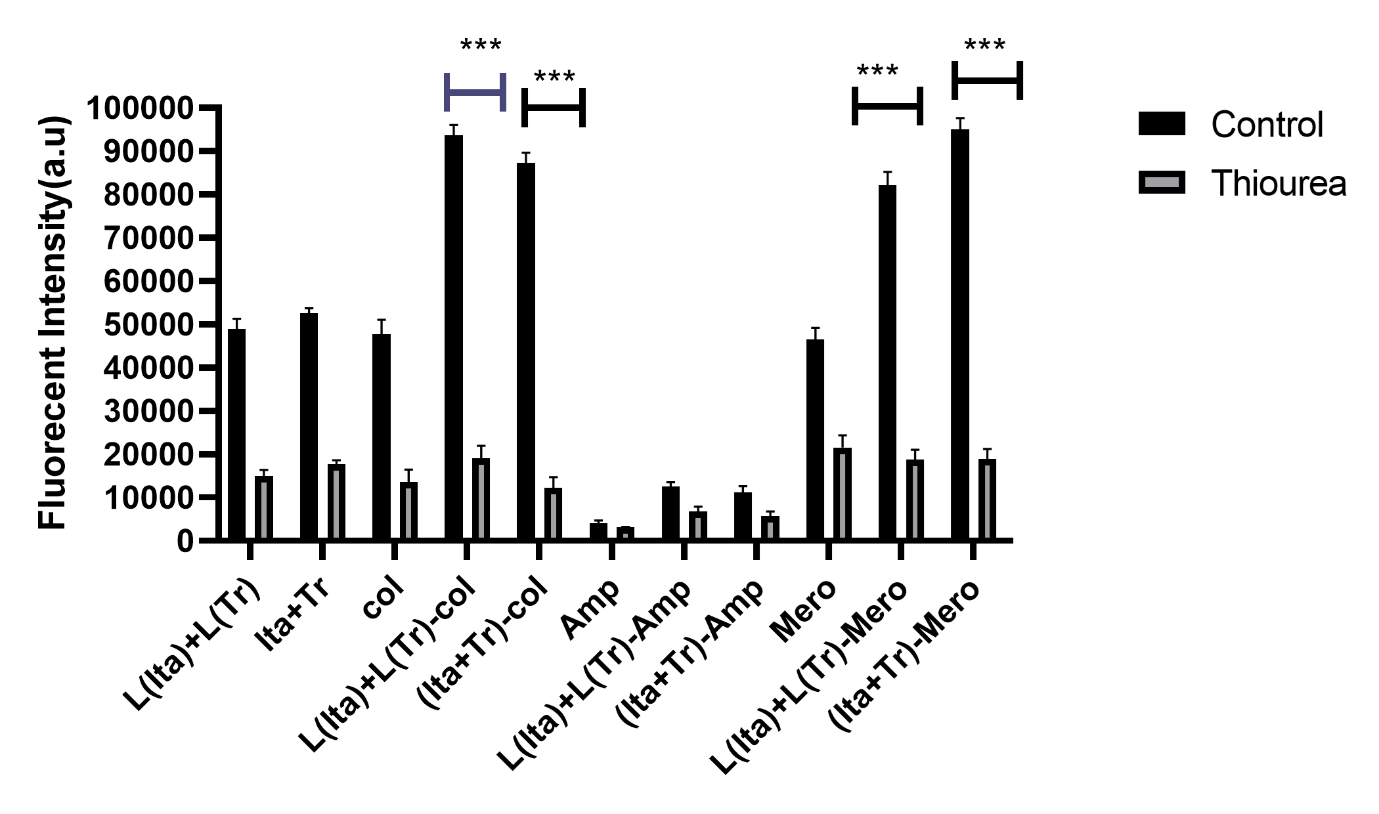


**Fig. S8.** Persister cells formation in the presence of thiourea (200mM) against 30X colistin, 40X Meropenem, 40X Ampicillin and 50µL Cell free supernatant and their combination. A) With CFS of *L. jensenii* B) With CFS of *L. fermentum* C) With Extracted and procured Itaconic anhydride (8µg/ml) and (-)-terpinen-4-ol (5 µg/ml) .The data is representative of three independent experiments. Bars represent the mean±SD. *p≤0.05; **p≤0.01; ***p≤0.001.

**
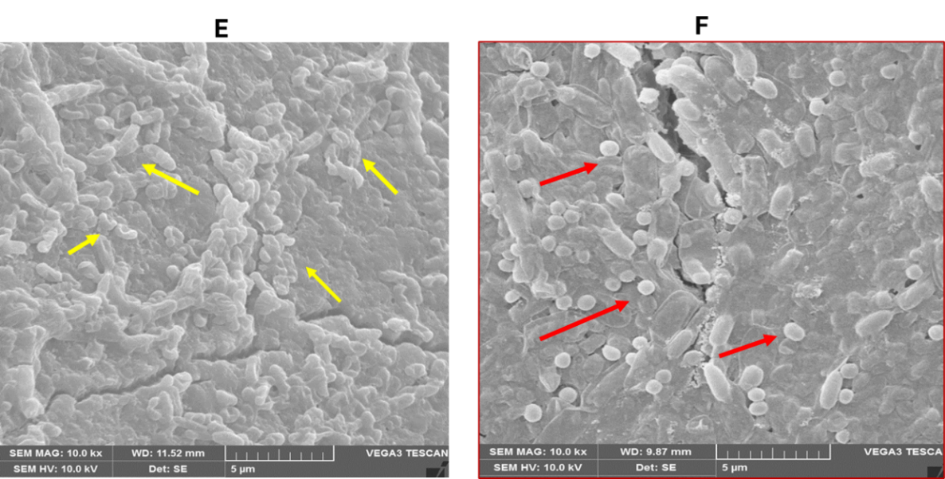

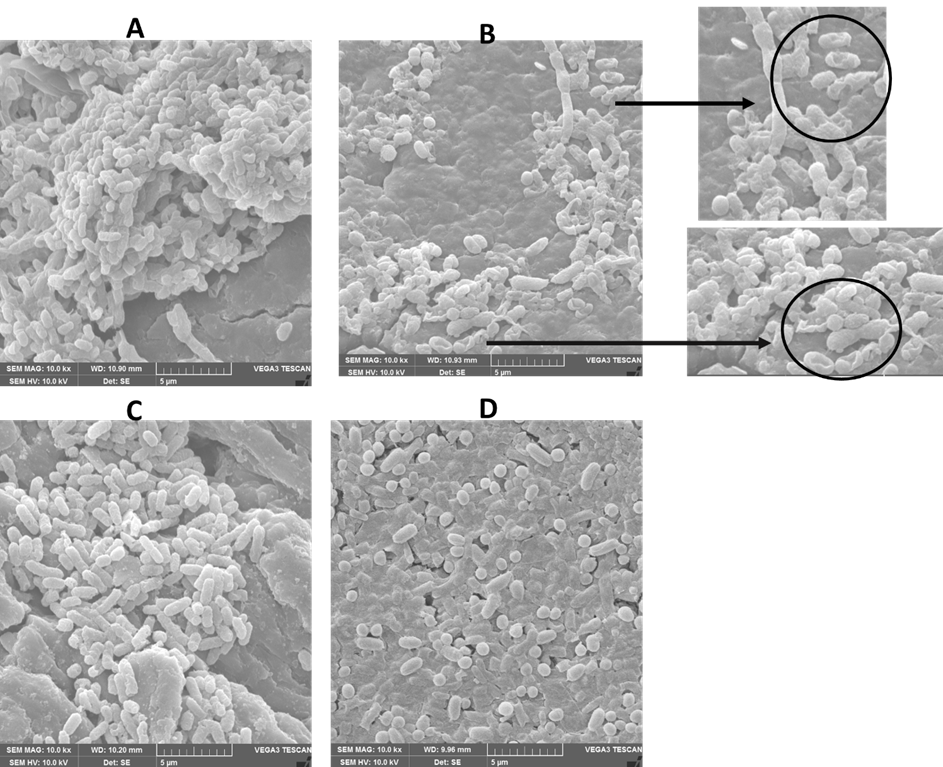
**

**Fig. S9:** Scanning Electron Microscopy (SEM) images illustrating the morphological characteristics of UPEC persister preformed biofilms after treatment with 30X colistin and 40X meropenem, with and without CFS. (A) colistin-derived persister biofilms (B), CFS-treated colistin-derived persister biofilms (C), meropenem-derived persister biofilms (D) CFS-treated meropenem-derived persister biofilms E), colistin-derived persister biofilms treated with a combination of extracted itaconic anhydride (8µg/ml) and (-)-terpinen-4-ol (5 µg/ml). The yellow arrow indicates persister cells showing surface roughness and disruptions in membrane integrity, indicating the impact of metabolite treatment. F) meropenem-derived persister biofilms treated with a combination of extracted itaconic anhydride (8µg/ml) and (-)-terpinen-4-ol (5 µg/ml). Red arrow indicates persister cells displaying altered morphology.


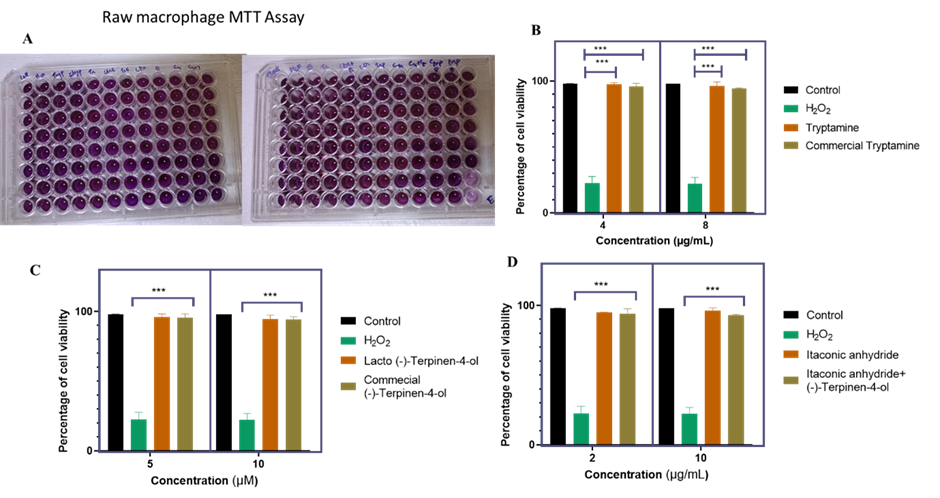


**Fig. S10. *In vitro* cell line toxicity studies for using Raw macrophages.** A) Plate showing MTT Assay B) For Tryptamine (4 µg/ml) C) (-)-terpinen-4-ol (5 µg/ml). D) itaconic anhydride (8µg/ml) p < 0.05, significant difference by ANOVA followed by Tukey's test.


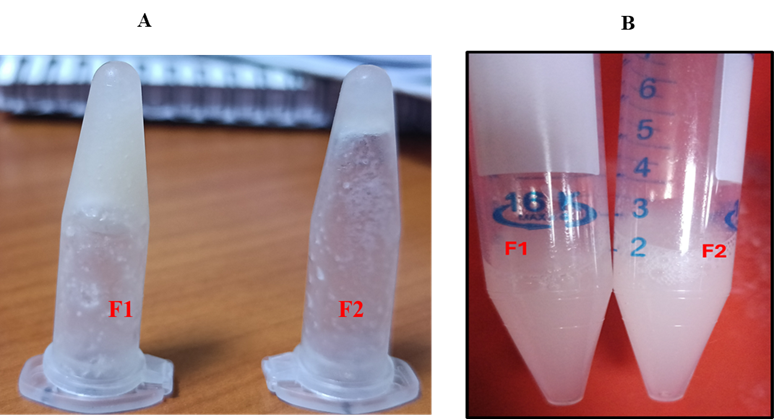


**Fig. S11**. Sol-Gel Transformation of the customized vaginal wash formulation using Poloxamer-407, where F1 is the *Lactobacillus* extracted metabolites incorporated in 18% Poloxamer 407 and F2 is the procured metabolites incorporated in 18% Poloxamer 407 A) Gel form at 37^0^C B) Solution form at below 20 ^0^ C.


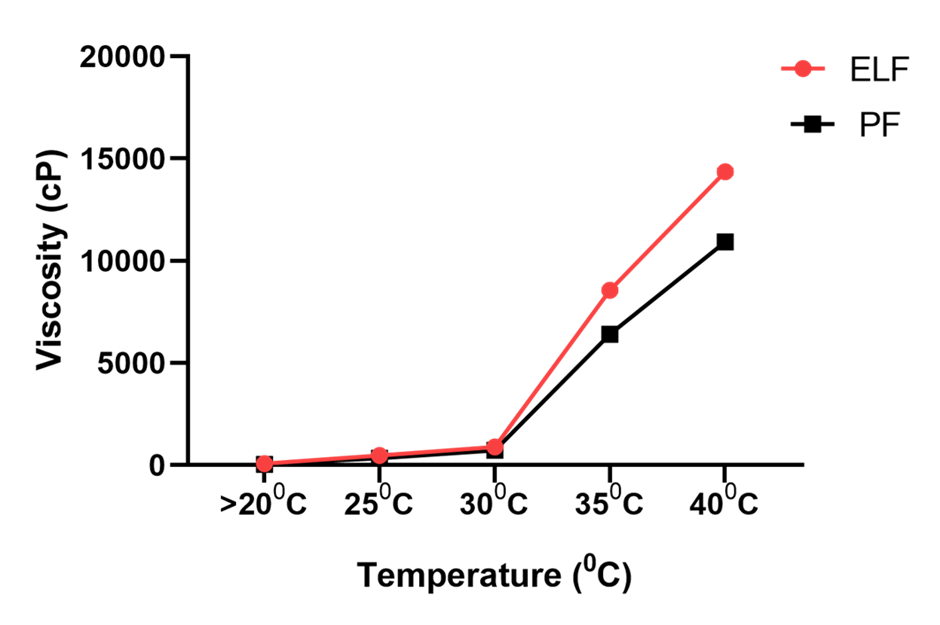


**Fig. S12**. Rheological analysis of the formulation to check the viscosity using Brookfield Viscometer (LVDV-II + Pro, Brookfield Engineering, USA) with spindle no. 64. ELF means Extracted metabolites from *Lactobacillus* incorporated in poloxamer 407, and PLF procured compounds incorporated in poloxamer 407.


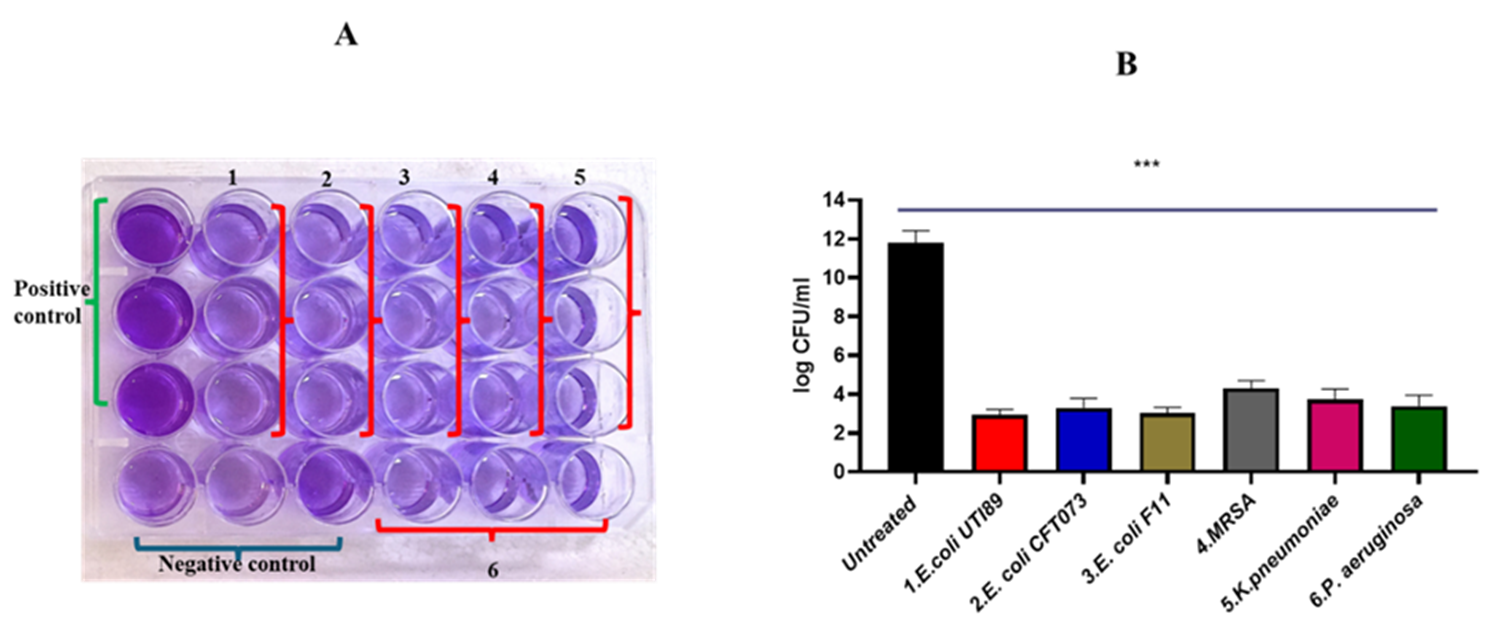


**Fig. S13** **. Stability Assessment of Customized *Lactobacillus*-Derived Metabolite Wash**. Crystal Violet (CV) assay (A) and Colony Forming Unit (B) count following the evaluation of the customized lactobacillus-derived wash against multiple clinical strains of bacteria. It includes wells for the Positive Control, indicating bacterial growth and biofilm formation in the absence of treatment, and the Negative Control, showing minimal bacterial growth and biofilm formation without exposure to the wash. The wells were inoculated with specific clinical strains: 1. *E. coli* UTI89, 2. *E. coli* CFT073, 3. *E. coli* F11, 4. Methicillin-Resistant *Staphylococcus aureus* (MRSA), 5. *Klebsiella pneumoniae* and 6. *Pseudomonas aeruginosa*. One tailed *t* test was performed to determine the significance * p<0.05, **p<0.01, ***p<0.001 n=3)


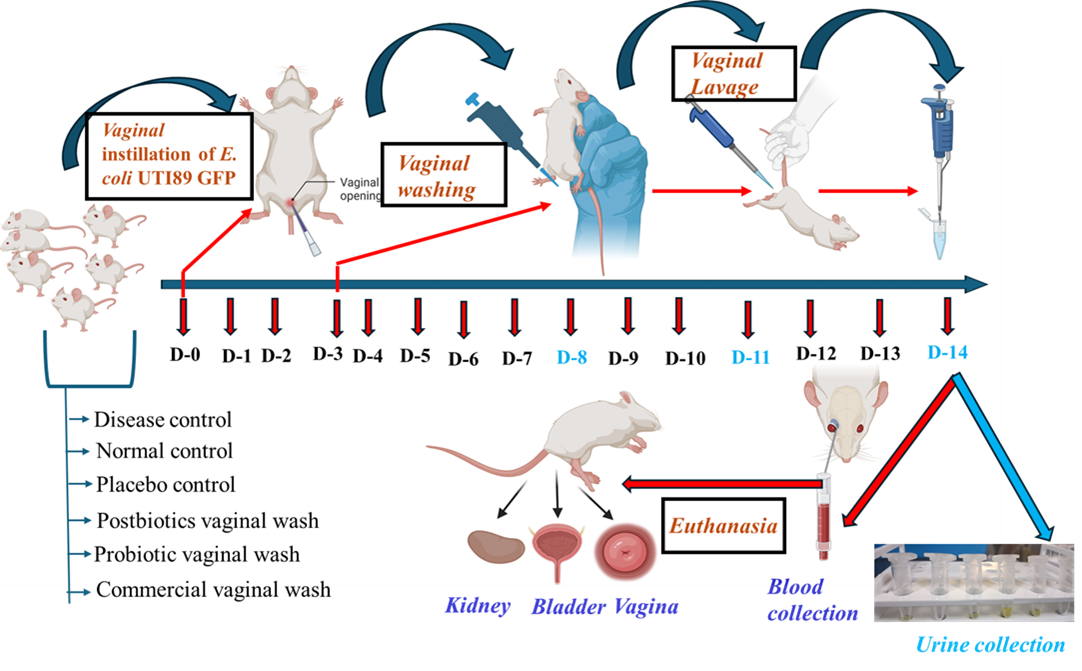


**Fig. S14:** Schematics of Animal Study


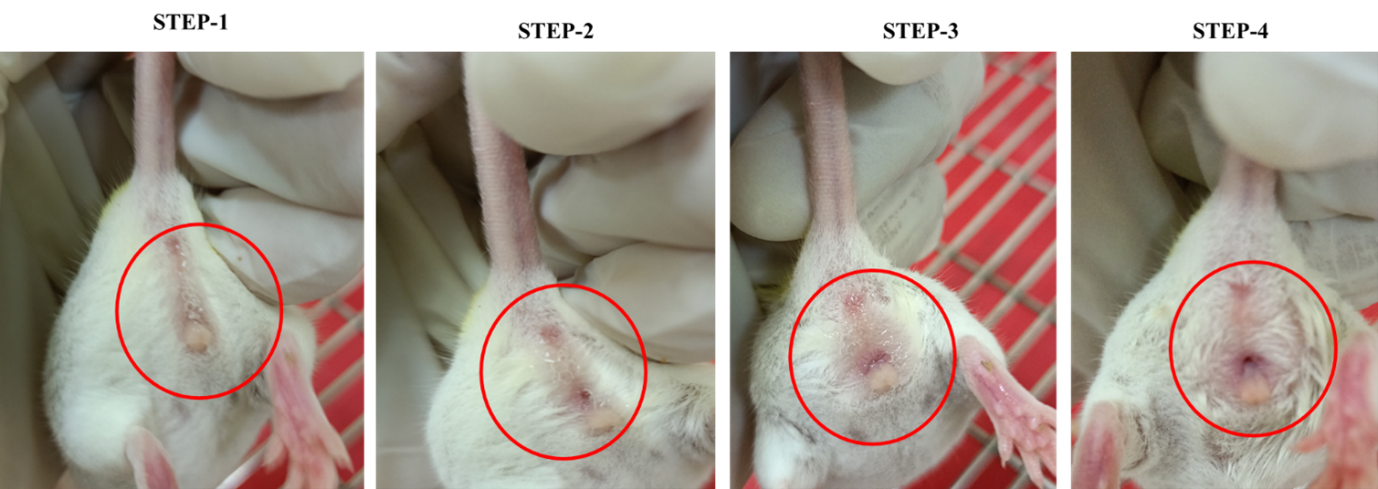


**Fig. S15** **Steps showing the washing of the vagina of BALB/c Mice with the formulation**. Step 1: A precise volume of 1 ml of the formulation is carefully poured from the perianal area, allowing it to flow into the vaginal opening. Step 2: The mouse is held in this position for two minutes to ensure adequate exposure to the formulation. Step 3: During this period, the formulation transitions into a gel-like consistency, which enhances its adherence and effectiveness. Step 4: After the two-minute wait, a gentle water wash is performed. A wash bottle is used to deliver the water, ensuring a thorough but gentle wash to remove the gel.


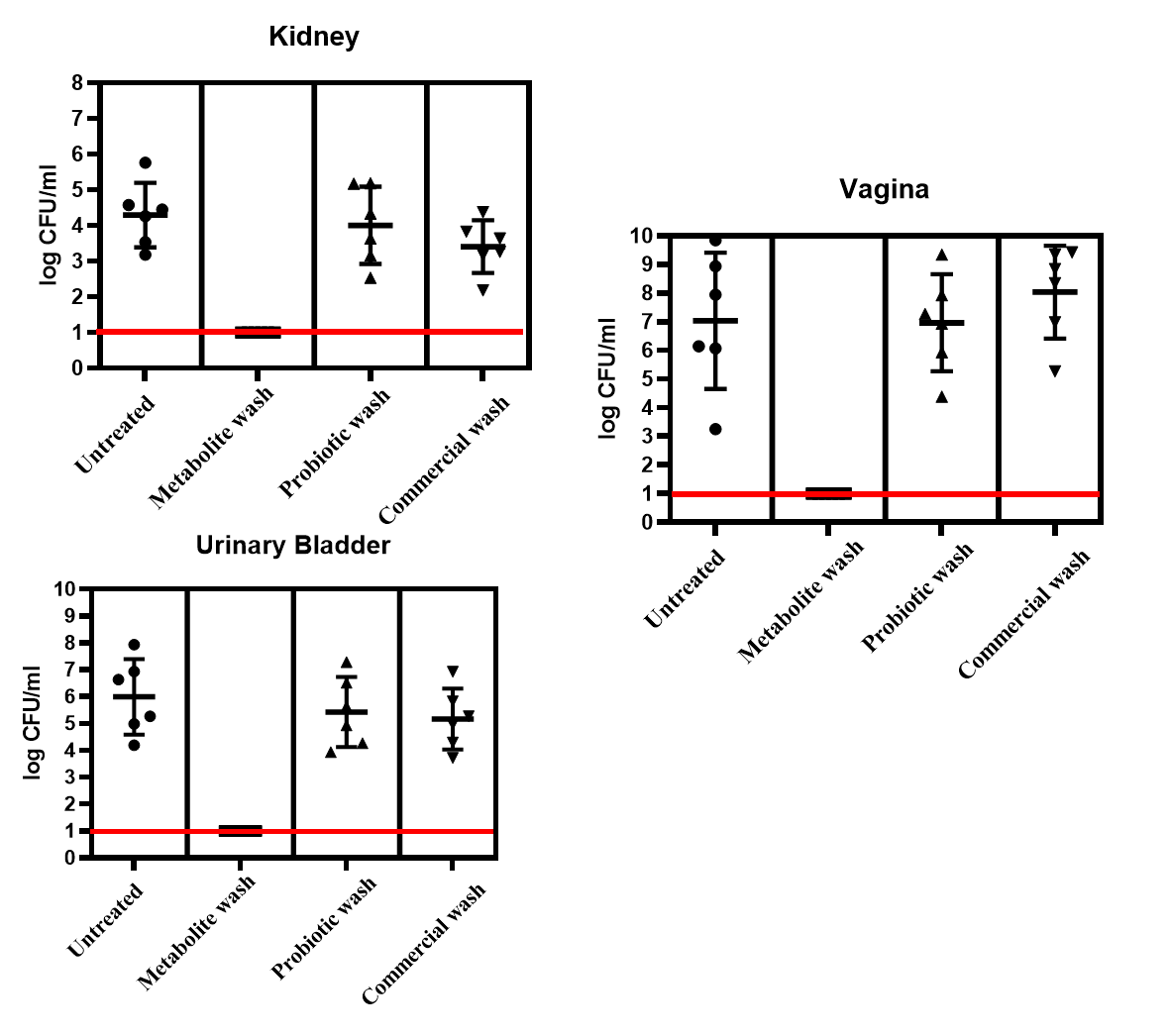


**Fig. S16** **Bacterial bioburdens in organs (Kidney, Vagina, and Urinary bladder)** Quantifications were performed in triplicate and represented as a mean ± standard error of the mean (SEM) (N= 3)

**Fig. S17 Serum creatinine Assay.** Normal serum creatinine values in BALB/c mice range from 0.08 to 0.11 mg/dL. Two samples got hemolyzed during blood collection time. Data represent the mean ± SD.
